# A study on the association between TP53 Arg72Pro, XRCC1 Arg399Gln, GSTP1 Ile105Val, and GSTM3 indel and the risk of cutaneous squamous cell carcinoma: a systematic review and meta-analysis

**DOI:** 10.1007/s12672-026-04433-2

**Published:** 2026-01-21

**Authors:** Tingting Zhang, Lixia Liu, Jihai Shi

**Affiliations:** 1 Affiliated Bayannur Clinical College of Inner Mongolia Medical University, Bayannur, 015000 Inner Mongolia Autonomous Region China; 2 Department of Dermatology, Bayannur Hospital, Bayannur, 015000 Inner Mongolia Autonomous Region China; 3 Department of Dermatology, The First Affiliated Hospital of Baotou Medical College, Inner Mongolia University of Science and Technology, Baotou, 014000 Inner Mongolia Autonomous Region China

**Keywords:** Cutaneous squamous cell carcinoma, Genetic polymorphisms, TP53, XRCC1, GSTP1, GSTM3

## Abstract

**Background:**

As one of the most frequent malignancies, cutaneous squamous cell carcinoma (cSCC) exhibits a rising occurrence, imposing a substantial burden on healthcare systems. This meta-analysis unveils the link to cSCC susceptibility of polymorphisms in Tumor Protein p53 (TP53 Arg72Pro, rs1042522), X-ray Repair Cross-Complementation Group 1 (XRCC1 Arg399Gln, rs25487), Glutathione S-Transferase Pi 1 (GSTP1 Ile105Val, rs1695), and the 3-bp insertion/deletion polymorphism in intron 6 of the Glutathione S-Transferase Mu 3 (GSTM3) gene.

**Methods:**

PubMed, Embase, Cochrane Library, as well as Web of Science were retrieved systematically to obtain pertinent case-control research until September 2024. The relations of TP53 Arg72Pro, XRCC1 Arg399Gln, GSTP1 Ile105Val, to the GSTM3 intron 6 insertion/deletion polymorphism (hereafter, GSTM3 indel) and cSCC risk were elucidated utilizing odds ratios (ORs) with 95% confidence intervals (CIs) in additive, dominant, recessive, homozygous, heterozygous, as well as allelic models. Heterogeneity was assessed using the Cochrane Q test and the I² statistic. To further explore potential sources of heterogeneity, subgroup analyses were conducted based on geographic region, sample size, source of controls, genotyping methods, and conformity with Hardy-Weinberg equilibrium. The robustness of the results was evaluated through sensitivity analyses. Publication bias was assessed using funnel plots and Egger’s test when ten or more eligible studies were included.

**Results:**

This meta-analysis revealed no significant association between the TP53 Arg72Pro, XRCC1 Arg399Gln, GSTP1 Ile105Val, and the GSTM3 indel and the risk of cSCC across all genetic models and the risk of cSCC across all genetic models. However, these gene polymorphisms exhibited substantial population heterogeneity and model-dependent associations with cSCC risk. Among the five genetic models, excluding the recessive model, TP53 Arg72Pro polymorphism was associated with an increased cSCC risk in Asian populations. XRCC1 Arg399Gln polymorphism was associated with elevated cSCC risk in European populations under the additive, allelic, and homozygous models, whereas it was associated with a decreased risk in North American populations. Notably, GSTP1 Ile105Val displayed a bidirectional effect in European populations: it was associated with reduced risk under the additive, allelic, and recessive models, but with increased risk under the homozygous model. The GSTM3 indel showed no significant association in any analysis.

**Conclusion:**

Our findings suggest that TP53 Arg72Pro, XRCC1 Arg399Gln, GSTP1 Ile105Val, and the GSTM3 indel are unlikely to be risk factors for cSCC. Further well-designed case-control studies are warranted to comprehensively assess the potential roles of these four polymorphisms in cSCC susceptibility.

**Supplementary Information:**

The online version contains supplementary material available at 10.1007/s12672-026-04433-2.

## Introduction

Cutaneous squamous cell carcinoma (cSCC), the second most prevalent skin cancer globally after basal cell carcinoma, is classified under the broader category of non-melanoma skin cancers [1]. According to the Global Burden of Disease Study 2017, the incidence of cSCC increased by an estimated 310% across 195 countries from 1990 to 2017, making it the most rapidly increasing cancer type among all malignancies tracked in the study [2]. The metastatic risk of cSCC is approximately 5%, with immunocompromised patients exhibiting a higher metastatic risk of 5% to 10% [3, 4]. The prognosis for the metastatic cSCC population is typically unfavorable, with five-year and ten-year survival rates of 25% − 35% and less than 10% [3]. The prevalence of cSCC is rising among global Caucasian populations, increasing its global prevalence and imposing a considerable strain on healthcare systems [5]. Therefore, it is pressing to identify specific biomarkers predictive of cSCC to facilitate early diagnosis and treatment, reduce incidence rates, and optimize healthcare resource utilization.

Many studies indicated the influence of various genetic polymorphisms on the susceptibility to cSCC [5–7]. Among these, mutations in the tumor suppressor gene Tumor Protein p53 (TP53) are the most frequently observed genetic alterations in cSCC [7]. The X-ray Repair Cross-Complementation Group 1 (XRCC1) gene plays a pivotal role in the base excision repair (BER) pathway, which is essential for the maintenance of genomic integrity [8]. Polymorphic variants of XRCC1 have been associated with reduced DNA repair efficiency, predisposing affected individuals to the accumulation of genetic damage, dysregulated cell proliferation, and eventual oncogenesis [9]. The Arg399Gln variant of XRCC1 is linked to various malignancies [10, 11]. Moreover, the glutathione S-transferase (GST) gene family, particularly Glutathione S-Transferase Pi 1 (GSTP1) and Glutathione S-Transferase Mu 3 (GSTM3), is known to be overexpressed in several preneoplastic and neoplastic skin tissues. These enzymes are critical in cellular detoxification, especially in neutralizing electrophilic byproducts arising from ultraviolet (UV) radiation-induced oxidative stress [12–14].

To elucidate the influence of genetic polymorphisms on cSCC, recent investigations have examined the associations of polymorphisms such as TP53 Arg72Pro (rs1042522), XRCC1 Arg399Gln (rs25487), GSTP1 Ile105Val (rs1695), and the GSTM3 intron 6 insertion/deletion polymorphism (hereafter, GSTM3 indel) [15] with disease susceptibility. About TP53 Arg72Pro, several studies have reported no significant association between this polymorphism and cSCC risk [16–23], whereas others suggest that TP53 Arg72Pro polymorphism may influence cSCC development and progression [24–27]. Concerning XRCC1 Arg399Gln, Surdu et al. [28] found the link of the homozygous variant genotype of XRCC1 Arg399Gln to an elevated cSCC risk, while two other studies reported a correlation between XRCC1 Arg399Gln polymorphism and lowered likelihood of squamous cell carcinoma (SCC) [10, 29]. Additionally, two further studies demonstrated no evident association [9, 30]. Multiple studies have linked GST polymorphisms to skin cancer susceptibility, with two studies reporting a relation of GST polymorphisms to the likelihood of non-melanoma skin cancer [31, 32], whereas Lira et al. [33] proved a relation of GST polymorphisms to the likelihood of cSCC. However, a study by Leite et al. [34] revealed no significant association between GSTP1 Ile105Val and the risk of developing cSCC.

Given the inconsistency in findings among previous studies, a meta-analysis was conducted to elucidate these associations.

## Methods

This meta-analysis was conducted in strict accordance with the Preferred Reporting Items for Systematic Reviews and Meta-Analyses (PRISMA) guidelines and the Meta-analysis Of Observational Studies in Epidemiology (MOOSE) reporting standards [35, 36]. The study protocol has been prospectively registered in the International Prospective Register of Systematic Reviews (CRD42024625949).

### Search strategy

PubMed, Embase, Cochrane Library, and Web of Science were retrieved until September 14, 2024. Only English studies were considered. MeSH terms and free-text keywords were employed: “Cutaneous squamous cell carcinoma”, “Squamous cell carcinoma of the skin”, “cSCC”, “Genes”, as well as “Polymorphism, Single Nucleotide”. Our strategy is detailed in Supplementary Table 1. To ensure comprehensiveness, manual screening of references from pertinent systematic reviews and meta-analyses was performed for additional qualifying studies.

### Eligibility criteria

Studies were incorporated if they (1) investigated the association between specific genetic polymorphisms and the risk of cSCC; (2) provided complete genotype distribution data for single nucleotide polymorphisms (SNPs) in both case and control groups; (3) employed a case-control study design; and (4) reported odds ratios (ORs) or sufficient data to compute them. If multiple studies analyzed overlapping datasets, the one with the most samples was included.

The studies were excluded if they (1) did not include patients with cSCC; (2) included individuals with cSCC in the control group instead of healthy controls; (3) had unavailable full texts; (4) were duplicate publications; (5) were conducted on non-human subjects; (6) has incomplete or erroneous data, such as incomplete case-control information or insufficient SNP genotype data; and (7) contained fewer than three studies on a specific SNP according to the inclusion criteria.

### Literature screening

All retrieved records were imported into EndNote 21.0 to remove duplicates. Two independent reviewers (Zhang and Liu) checked pertinent titles and abstracts as per the eligibility criteria. Irrelevant records were eliminated at this stage. Full-text articles of potentially eligible studies were retrieved either through institutional access or by contacting the corresponding authors directly. A second round of screening was conducted based on full-text review to finalize inclusion. Disagreements were resolved through discussion or consultation with a third reviewer (Shi). Final decisions were made by consensus among all investigators.

### Data extraction

Two reviewers (Zhang and Liu) retrieved data independently using Microsoft Excel 2016. Any discrepancies were discussed and, if necessary, resolved with the involvement of a third investigator (Shi) to ensure consensus. The extracted data were gene loci, first author, publication year, region, ethnicity, source of controls, genotyping approach, as well as sample sizes.

### Quality assessment

Two independent reviewers (Zhang and Liu) rated the study quality using the Newcastle-Ottawa Scale (NOS) across three domains and eight items: selection domain (case and control definition and selection); comparability domain (comparability of cases and controls); exposure domain (exposure ascertainment, case and control ascertainment, and non-response rate). The “comparability” criterion was allocated a maximum score of two, with the rest rated at one each. The total score was 0–9, with ≥ 6 denoting high quality and ≤ 5 indicating low quality ( Supplementary Table 2).

### Statistical analysis

SPSS 26.0 and Stata 15.0 were leveraged for statistical analysis. The quantitative synthesis was performed using SPSS 26.0, while heterogeneity test, subgroup and sensitivity analysis, and publication bias evaluation were conducted via Stata 15.0. This study employed the additive, allelic, homozygous, heterozygous, dominant, and recessive models for the quantitative synthesis of eligible studies. The relation of genetic polymorphisms to cSCC risk was examined utilizing OR and 95% CI. A two-sided test was undertaken with a significance level of α = 0.05. Statistical heterogeneity was detected through the Cochrane Q test and I² statistic, with *P* < 0.10 or I²>50% indicating significant variation. A random-effects model was adopted in case of notable heterogeneity; otherwise, a fixed-effects model was employed. The reliability of results was verified via sensitivity analysis To further explore this heterogeneity, subgroup analyses were conducted based on geographic region, sample size, source of controls, genotyping method, and conformity with Hardy-Weinberg equilibrium. In cases of 10 or more studies, funnel plots and Egger’s test helped with publication bias identification. *p* < 0.05 denoted statistical significance and publication bias.

## Results

### Literature search and selection process

**Fig. 1:**
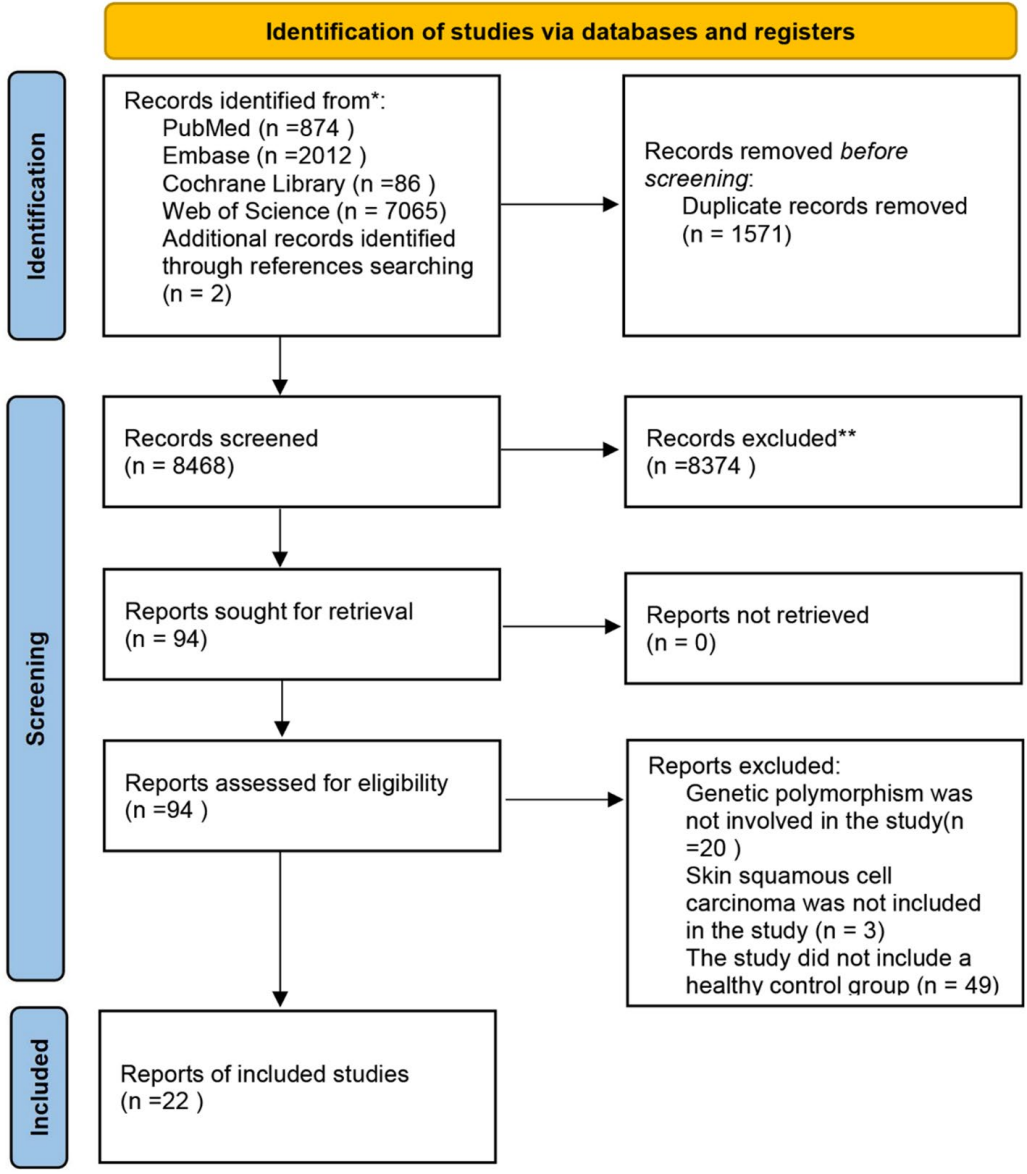
Screening flowchart

10,037 articles were retrieved through the literature search. After the removal of 1,549 duplicates, 8,396 records were excluded after title and abstract screening. The remaining articles underwent full-text review as per predefined eligibility criteria. Additionally, reference lists of eligible studies and relevant review articles were manually screened to identify any additional pertinent publications. Ultimately, 22 studies met the inclusion criteria and were incorporated into the meta-analysis (Fig. 1).

### Characteristics and quality assessment of included studies

The 22 included studies originated from 16 countries: the United Kingdom, the United States, France, South Korea, Japan, the Netherlands, Sweden, Greece, India, Brazil, Mexico, Italy, Australia, Hungary, Romania, and Slovakia. In total, the studies comprised 2,617 cSCC cases and 5,807 controls. Eight genotyping techniques were reported, including polymerase chain reaction (PCR), PCR with sequence-specific primers (PCR-SSP), PCR-denaturing gradient gel electrophoresis (PCR-DGGE), allele-specific PCR sequencing (AS-PCR-seq), nested PCR-pyrosequencing (nPCR-PSQ), TaqMan genotyping assay, PCR-restriction fragment length polymorphism (PCR-RFLP), and nested PCR-cDNA sequencing (nPCR-cDNA-seq). The analysis focused on four genetic loci: TP53 Arg72Pro, XRCC1 Arg399Gln, GSTP1 Ile105Val, and the GSTM3 indel. The characteristics are illustrated in Table 1. The quality assessment yielded scores greater than 6, indicating high-quality research (Table 1).

**Table 1:** Basic characteristics of the studies included in the Meta-Analysis

Gene	First author	Year	Region	Race	Reference source	Assay	Case			Control			NOS score
							AA	AB	BB	AA	AB	BB	
TP53 Arg72Pro	Marshall	2000	UK	Caucasian	PB	PCR-SSP	18	14	2	39	39	6	8
	Dokianakis	2000	Greece	Caucasian	PB	PCR-SSP	2	1	0	12	41	6	6
	Bastiaens	2001	The Netherlands	Caucasian	HB	PCR-SSP	41	40	6	75	72	10	7
	Cairey-Remonnay	2002	France	Caucasian	PB	PCR-DGGE	50	16	4	17	7	5	7
	McGregor	2002	UK	Caucasian	HB	AS-PCR-seq	74	35	0	85	66	5	8
	Humbey	2003	France	Caucasian	HB	PCR-DGGE	19	12	0	15	7	0	6
	Gustafsson	2004	Sweden	Caucasian	PB	nPCR-PSQ	30	19	5	62	31	3	8
	Han	2006	USA	Caucasian	PB	TaqMan	151	104	17	474	297	45	9
	Bendesky	2007	Mexico	Other	HB	PCR-RFLP	18	21	3	126	94	18	7
	Almquist	2011	USA	Caucasian	PB	PCR-RFLP	366	220	37	446	274	47	9
	Loeb	2012	USA	Caucasian	PB	nPCR-cDNA-seq	35	16	4	17	19	5	9
	Pandith	2012	India	Asia	HB	PCR-RFLP	25	62	19	90	78	32	8
XRCC1 Arg399Gln	Nelson	2002	USA	Caucasian	PB	PCR-RFLP	108	113	25	175	185	71	9
	Han	2004	USA	Mixed	PB	TaqMan	128	112	33	345	351	119	9
	Kang	2007	South Korea	Asia	HB	PCR-RFLP	48	38	11	108	85	12	8
	Chiyomaru	2012	Japan	Asia	HB	PCR-RFLP	15	9	3	51	21	21	7
	Surdu	2014	Mixed	Caucasian	HB	TaqMan	21	34	14	209	243	63	9
GSTP1 Ile105Val	Ramsay	2001	UK	Caucasian	HB	PCR-RFLP	10	10	0	53	71	17	8
	Fryer	2004	Australia	Caucasian	HB	PCR-RFLP	59	51	18	95	90	25	9
	Lira	2006	Italy	Caucasian	HB	PCR-RFLP	26	20	1	58	48	24	9
	Leite	2007	Brazil	Mixed	PB	PCR-RFLP	14	13	2	60	46	18	8
GSTM3 indel	Ramsay	2001	UK	Caucasian	HB	PCR	15	4	1	95	44	2	8
	Fryer	2004	UK	Caucasian	HB	PCR	91	35	2	149	55	4	9
	Lira	2006	Italy	Caucasian	HB	PCR	34	10	3	86	37	6	9

*PB* population-based, *HB* hospital-based, *NOS* Newcastle-Ottawa Scale, *AA* TP53 Arg/Arg, XRCC1 Arg/Arg, GSTP1 Ile/Ile, GSTM3 A/A, *AB*, TP53 Arg/Pro, XRCC1 Arg/Gln, GSTP1 Ile/Val, GSTM3 A/B; *BB*, TP53 Pro/Pro, XRCC1 Gln/Gln, GSTP1 Val/Val and GSTM3 B/B, *PCR* polymerase chain reaction, *PCR-SSP* PCR with sequence-specific primers, *PCR-DGGE* PCR-denaturing gradient gel electrophoresis, *AS-PCR-seq* allele-specific PCR sequencing, *nPCR-PSQ* nested PCR-pyrosequencing, *PCR-RFLP* PCR-restriction fragment length polymorphism, *nPCR-cDNA-seq* nested PCR-cDNA sequencing

### Meta-analysis results

#### Summary of the link of genetic polymorphisms to susceptibility to cSCC

##### Association of TP53 Arg72Pro polymorphism with susceptibility to cSCC

**Fig. 2:**
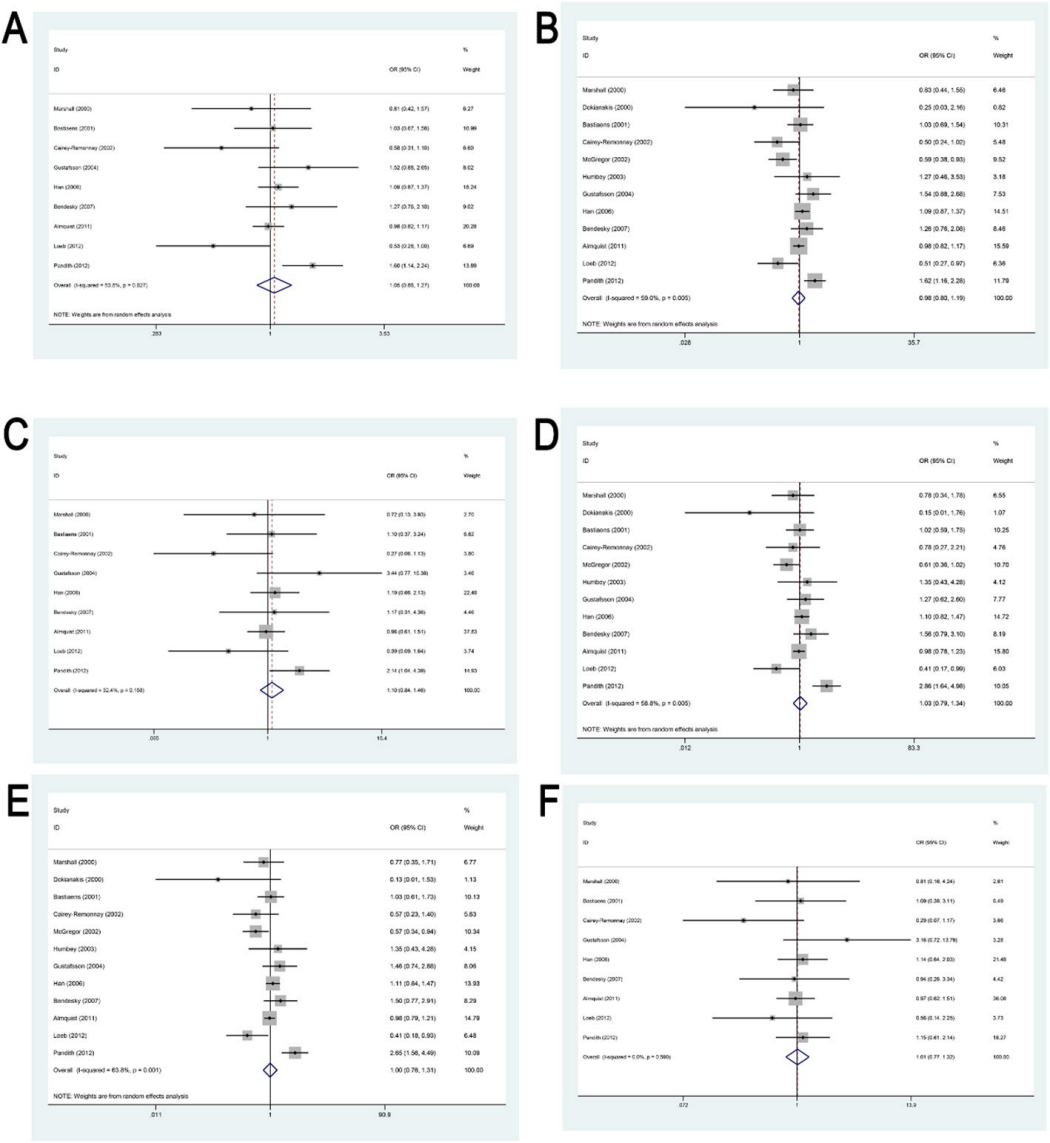
Forest Plot Demonstrating the Association Between TP53 Arg72Pro Polymorphism and Susceptibility to cSCC. **A**. Forest plot showing the association between TP53 Arg72Pro polymorphism and susceptibility to cSCC under the additive model. **B**. Forest plot showing the association between TP53 Arg72Pro polymorphism and susceptibility to cSCC under the allele model. **C**. Forest plot showing the association between TP53 Arg72Pro polymorphism and susceptibility to cSCC under the homozygous model. **D**. Forest plot showing the association between TP53 Arg72Pro polymorphism and susceptibility to cSCC under the heterozygous model. **E**. Forest plot showing the association between TP53 Arg72Pro polymorphism and susceptibility to cSCC under the dominant model. **F**. Forest plot showing the association between TP53 Arg72Pro polymorphism and susceptibility to cSCC under the recessive model

The association and heterogeneity analyses of this meta-analysis are summarized in Table 2 and Supplementary Table 3. A total of twelve studies investigated the relationship between this polymorphism and susceptibility to cSCC. In both the homozygous and recessive genetic models, no significant inter-study heterogeneity was observed; therefore, fixed-effect models were applied. Conversely, significant heterogeneity was detected in the remaining four models, warranting the use of random-effect models. Across all six genetic models, our statistical analyses did not demonstrate a significant association between the TP53 Arg72Pro polymorphism and an increased risk of cSCC (Fig. 2; Table 2).

**Table 2:** Main results of the Meta-analysis examining the associations between TP53 Arg72Pro, XRCC1 Arg399Gln, GSTP1 Ile105Val, and the GSTM3 indel and cSCC risk across different genetic models

SNPs	Genetic model	Number	Test of association		Test of heterogeneity		
			OR (95 CI)	P-value	I2 (%)	Ph	Model
TP53 Arg72Pro	Additive	9	1.046( 0.863–1.267)	0.647	53.8	0.027	R
	Allelic	12	0.975( 0.797–1.193)	0.806	59.0	0.005	R
	Homozygous	9	1.103(0.835–1.457)	0.489	32.4	0.158	F
	Heterozygous	12	1.030(0.789–1.344)	0.831	58.8	0.005	R
	Dominant	12	0.997(0.760–1.309)	0.984	63.8	0.001	R
	Recessive	9	1.010(0.774–1.319)	0.940	0.0	0.590	F
XRCC1 Arg399Gln	Additive	5	0.994(0.795–1.244)	0.960	61.1	0.036	R
	Allelic	5	0.989(0.782–1.250)	0.923	62.4	0.031	R
	Homozygous	5	0.994(0.561–1.762)	0.983	70.7	0.009	R
	Heterozygous	5	0.986(0.817–1.189)	0.881	0.0	0.583	F
	Dominant	5	0.947(0.794–1.130)	0.546	18.7	0.296	F
	Recessive	5	0.967(0.572–1.633)	0.899	69.4	0.011	R
GSTP1 Ile105Val	Additive	3	0.867(0.677–1.109)	0.256	48.5	0.144	F
	Allelic	4	0.818(0.642–1.043)	0.105	45.4	0.139	F
	Homozygous	3	1.496(0.387–5.788)	0.560	65.0	0.057	R
	Heterozygous	4	0.933(0.669–1.301)	0.684	0.0	0.899	F
	Dominant	4	0.844(0.615–1.158)	0.292	0.0	0.658	F
	Recessive	3	0.477(0.119–1.913)	0.296	68.6	0.041	R
GSTM3 indel	Additive	3	0.948(0.680–1.321)	0.752	0.0	0.919	F
	Allelic	3	0.948(0.680–1.321)	0.752	0.0	0.913	F
	Homozygous	3	1.270(0.464–3.479)	0.642	0.0	0.677	F
	Heterozygous	3	0.877(0.589–1.305)	0.517	0.0	0.513	F
	Dominant	3	0.903(0.618–1.320)	0.599	0.0	0.701	F
	Recessive	3	1.362( 0.501–3.707)	0.545	0.0	0.612	F

*SNP* single nucleotide polymorphism, *N* not available, *OR* odds ratio, *CI* confidence interval, *F* fixed effect model, *R* random-effect model, *Ph* P-value of heterogeneity

In subgroup analyses stratified by geographic region, no significant association was observed between the TP53 Arg72Pro variant and cSCC risk among Asian populations under the recessive model. However, significant associations were identified under the other five genetic models (additive: OR = 1.599, 95% CI 1.141–2.240, *P* = 0.006; allelic: OR = 1.622, 95% CI 1.156–2.276, *P* = 0.005; homozygous: OR = 2.137, 95% CI 1.040–4.392, *P* = 0.039; heterozygous: OR = 2.862, 95% CI 1.644–4.984, *P* < 0.001; dominant: OR = 2.651, 95% CI 1.564–4.494, *P* < 0.001). In contrast, no significant association was identified between TP53 Arg72Pro and cSCC in European or North American populations (*P* > 0.05 across all models).

Subgroup analysis based on control source indicated that hospital-based studies showed a significant association between the TP53 Arg72Pro variant and increased cSCC risk under the additive model (OR = 1.319, 95% CI 1.009–1.725, *P* = 0.043), while no significant associations were observed in the remaining five models (*P* > 0.05). In contrast, population-based studies did not identify any significant association between the polymorphism and cSCC risk across all genetic models (*P* > 0.05).

When stratified by conformity to Hardy-Weinberg equilibrium (HWE), studies not in HWE demonstrated significant associations under the additive and homozygous models (additive: OR = 1.599, 95% CI 1.141–2.240, *P* = 0.006; homozygous: OR = 2.137, 95% CI 1.040–4.392, *P* = 0.039). However, the other four models showed no statistically significant associations (*P* > 0.05).

Subgroup analyses based on sample size and genotyping method did not reveal any significant association between TP53 Arg72Pro polymorphism and cSCC risk across the six genetic models examined (*P* > 0.05) (Supplementary Table 3).

#### Association of XRCC1 Arg399Gln polymorphism with susceptibility to cSCC

**Fig. 3:**
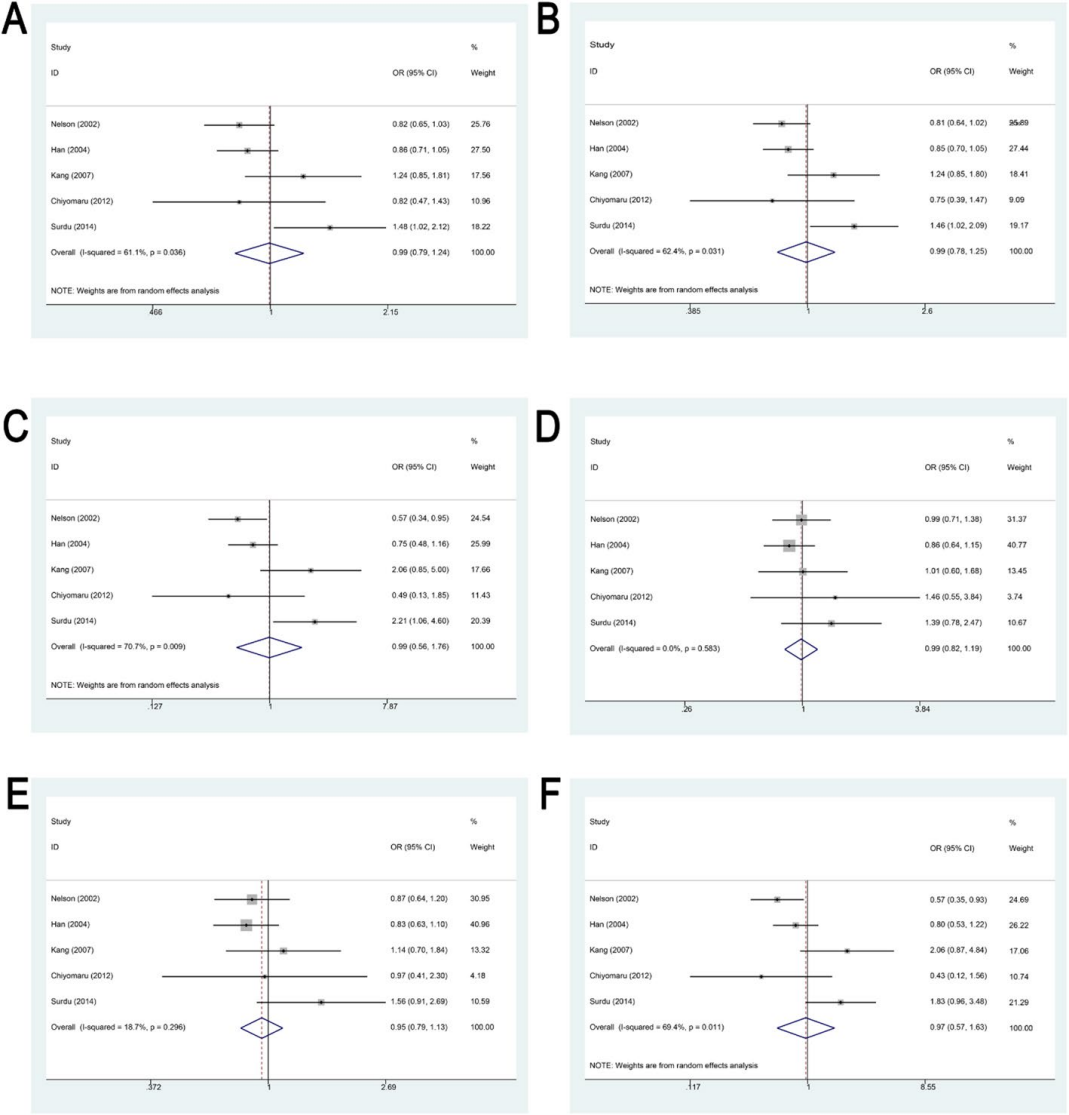
Forest Plot Demonstrating the Association Between XRCC1 Arg399Gln Polymorphism and Susceptibility to cSCC. **A**. Forest plot showing the association between XRCC1 Arg399Gln polymorphism and susceptibility to cSCC under the additive model. **B**. Forest plot showing the association between XRCC1 Arg399Gln polymorphism and susceptibility to cSCC under the allele model. **C**. Forest plot showing the association between XRCC1 Arg399Gln polymorphism and susceptibility to cSCC under the homozygous model. **D**. Forest plot showing the association between XRCC1 Arg399Gln polymorphism and susceptibility to cSCC under the heterozygous model. **E**. Forest plot showing the association between XRCC1 Arg399Gln polymorphism and susceptibility to cSCC under the dominant model. **F**. Forest plot showing the association between XRCC1 Arg399Gln polymorphism and susceptibility to cSCC under the recessive model

Five studies evaluated the potential association between the XRCC1 Arg399Gln polymorphism and cSCC susceptibility. Statistical analysis indicated no significant association across all genetic models (*P* > 0.05), as illustrated in Fig. 3; Table 2.

Geographic subgroup analysis showed that among European populations, the XRCC1 Arg399Gln variant was significantly associated with increased cSCC risk under the additive, allelic, and homozygous models (additive: OR = 1.475, 95% CI 1.024–2.124, *P* = 0.037; allelic: OR = 1.461, 95% CI 1.021–2.091, *P* = 0.038; homozygous: OR = 2.212, 95% CI 1.063–4.602, *P* = 0.034). No significant associations were detected under the remaining three models (*P* > 0.05). Among North American populations, no significant associations were observed under the heterozygous and dominant models (*P* > 0.05). However, the variant was inversely associated with cSCC risk in the other four models (additive: OR = 0.843, 95% CI 0.726–0.980, *P* = 0.026; allelic: OR = 0.835, 95% CI 0.716–0.975, *P* = 0.022; homozygous: OR = 0.668, 95% CI 0.479–0.931, *P* = 0.017; recessive: OR = 0.697, 95% CI 0.503–0.966, *P* = 0.030). No significant associations were observed among Asian populations in any genetic model (*P* > 0.05).

Subgroup analysis by control source revealed that population-based studies did not show significant associations under the heterozygous and dominant models (*P* > 0.05). However, inverse associations with cSCC risk were observed under the remaining four models (additive: OR = 0.843, 95% CI 0.726–0.980, *P* = 0.026; allelic: OR = 0.835, 95% CI 0.716–0.975, *P* = 0.022; homozygous: OR = 0.668, 95% CI 0.479–0.931, *P* = 0.017; recessive: OR = 0.697, 95% CI 0.503–0.966, *P* = 0.030).

Subgroup analyses stratified by sample size and genotyping method did not identify any significant associations between XRCC1 Arg399Gln polymorphism and cSCC across all six genetic models (*P* > 0.05; Supplementary Table 3).

#### Association of GSTP1 Ile105Val polymorphism with susceptibility to cSCC

**Fig. 4:**
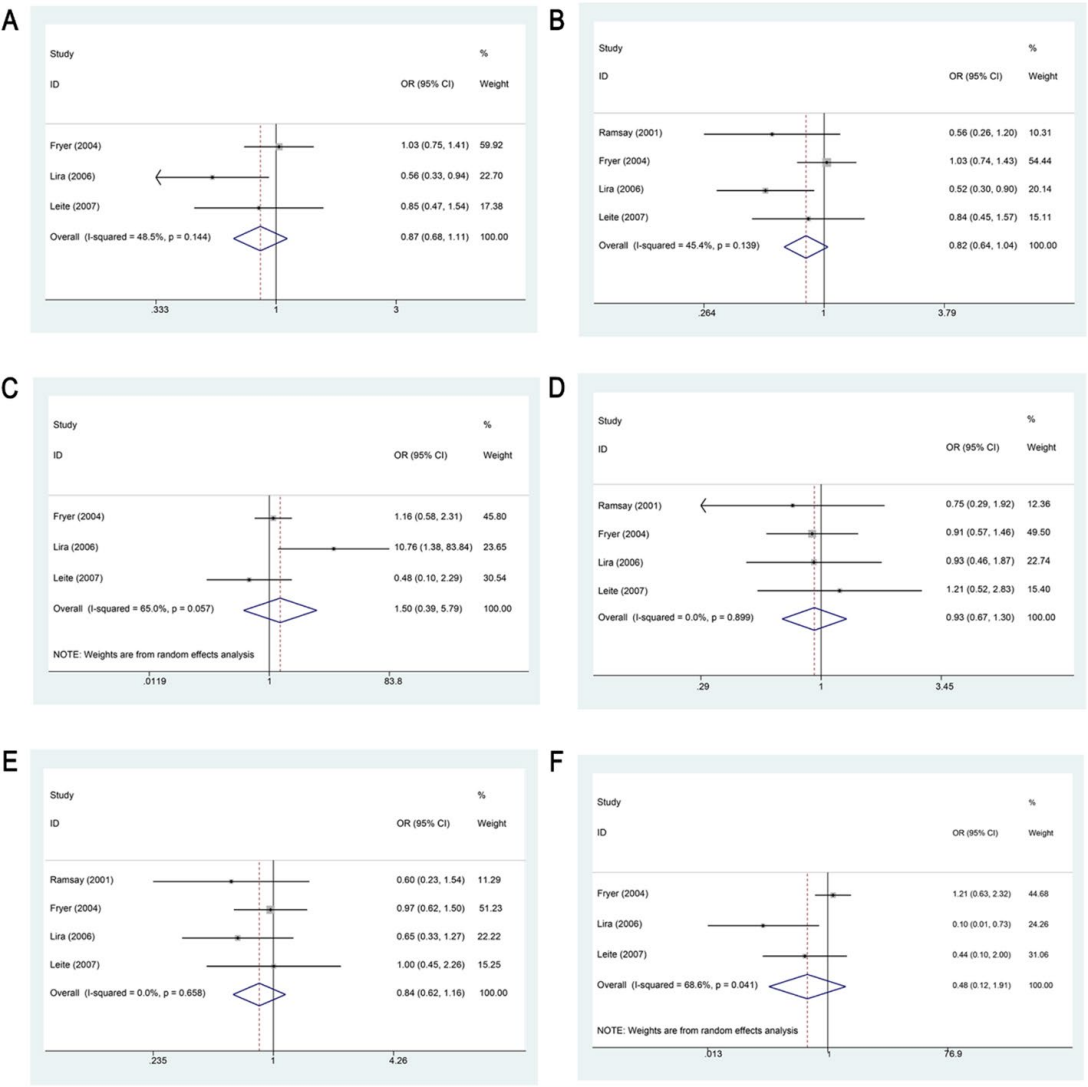
Forest Plot Demonstrating the Association Between GSTP1 Ile105Val Polymorphism and Susceptibility to cSCC. **A**. Forest plot showing the association between GSTP1 Ile105Val polymorphism and susceptibility to cSCC under the additive model. **B**. Forest plot showing the association between GSTP1 Ile105Val polymorphism and susceptibility to cSCC under the allele model. **C**. Forest plot showing the association between GSTP1 Ile105Val polymorphism and susceptibility to cSCC under the homozygous model. **D**. Forest plot showing the association between GSTP1 Ile105Val polymorphism and susceptibility to cSCC under the heterozygous model. **E**. Forest plot showing the association between GSTP1 Ile105Val polymorphism and susceptibility to cSCC under the dominant model. **F**. Forest plot showing the association between GSTP1 Ile105Val polymorphism and susceptibility to cSCC under the recessive model

Four studies examined the association between GSTP1 Ile105Val polymorphism and cSCC risk. Statistical analysis found no significant association between the variant and cSCC risk in any genetic model (*P* > 0.05), as depicted in Fig. 4; Table 2.

Regional subgroup analysis revealed that in European populations, the GSTP1 Ile105Val polymorphism was significantly associated with decreased cSCC risk under the additive, allelic, and recessive models (additive: OR = 0.558, 95% CI 0.332–0.937, *P* = 0.027; allelic: OR = 0.535, 95% CI 0.345–0.831, *P* = 0.005; recessive: OR = 0.096, 95% CI 0.013–0.720, *P* = 0.023). In contrast, under the homozygous model, the polymorphism was significantly associated with increased risk (OR = 10.759, 95% CI 1.381–83.829, *P* = 0.023). No significant associations were identified under the heterozygous or dominant models (*P* > 0.05). A subgroup analysis based on sample size revealed that in studies with a sample size of less than 200, the GSTP1 Ile105Val polymorphism was negatively related to susceptibility to cSCC under the allelic model (OR = 0.621, 95% CI 0.434–0.890, *P* = 0.009). No significant associations were observed under other genetic models. Furthermore, subgroup analysis by the source of controls showed no significant association between GSTP1 Ile105Val and cSCC risk under any genetic model (*P* > 0.05; Supplementary Table 3).

##### Association of the GSTM3 indel with susceptibility to cSCC

**Fig. 5:**
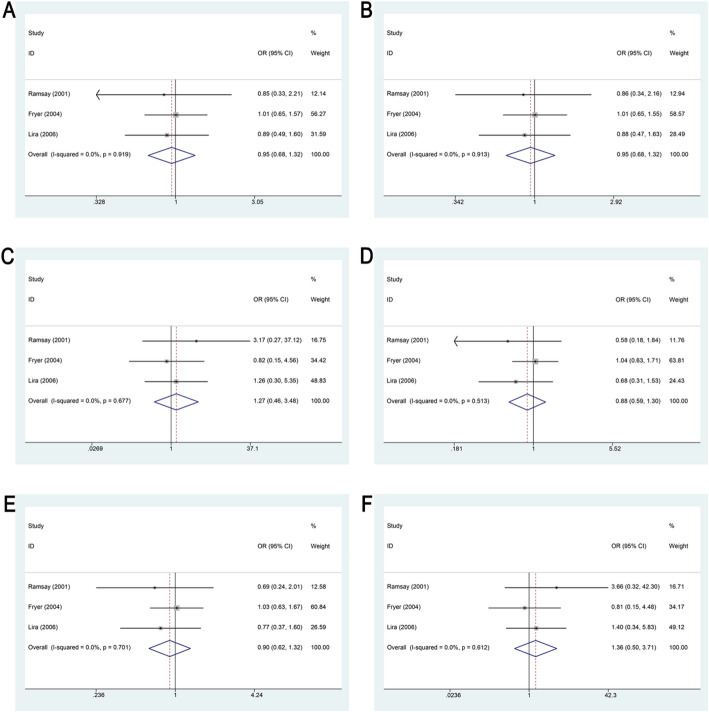
Forest Plot Demonstrating the Association Between the GSTM3 indel and Susceptibility to cSCC. **A**. Forest plot showing the association between the GSTM3 indel and susceptibility to cSCC under the additive model. **B**. Forest plot showing the association between the GSTM3 indel and susceptibility to cSCC under the allele model. **C**. Forest plot showing the association between the GSTM3 indel and susceptibility to cSCC under the homozygous model. **D**. Forest plot showing the association between the GSTM3 indel and susceptibility to cSCC under the heterozygous model. **E**. Forest plot showing the association between the GSTM3 indel and susceptibility to cSCC under the dominant model. **F**. Forest plot showing the association between the GSTM3 indel and susceptibility to cSCC under the recessive model

Three studies assessed the relationship between the GSTM3 indel and cSCC risk. Across all genetic models, no statistically significant associations were detected (*P* > 0.05), as illustrated in Fig. 5; Table 2. Subgroup analysis based on sample size also did not reveal any significant associations (*P* > 0.05; Supplementary Table 3).

### Heterogeneity analysis

For the TP53 Arg72Pro polymorphism, no significant inter-study heterogeneity was noted under either the homozygous or recessive genetic models, while the remaining models exhibited significant heterogeneity. For XRCC1 Arg399Gln, heterogeneity was minimal under the heterozygous and dominant models but was significant under the other four models. The GSTP1 Ile105Val polymorphism demonstrated significant heterogeneity under the homozygous and recessive models only. No heterogeneity was identified for the GSTM3 indel across any model (Table 2).

To identify potential sources of heterogeneity, subgroup analyses were conducted based on geographic region, sample size, control source, genotyping method, and HWE conformity (Supplementary Table 3). Control source may account for heterogeneity in the additive model for TP53 Arg72Pro (population-based: I²=49.0%, Pₕ=0.081; hospital-based: I²=20.8%, Pₕ=0.283). Regarding XRCC1 Arg399Gln, the control source also likely contributed to heterogeneity in the additive and allelic models. Specifically, population-based studies showed no heterogeneity in the additive (I²=0.0%, Pₕ=0.729) or allelic models (I²=0.0%, Pₕ=0.739), while hospital-based studies exhibited only mild heterogeneity (additive: I²=32.8%, Pₕ=0.226; allelic: I²=32.5%, Pₕ=0.227).

### Sensitivity analysis and publication bias

To assess the robustness of the findings, sensitivity analyses were conducted by sequentially excluding individual studies to evaluate their impact on the overall effect estimates. The results remained consistent throughout, indicating no substantial variation attributable to any single study (Figs. S1, S2, S3, and S4). Potential publication bias was assessed in analyses that included more than ten studies, using both funnel plot visualization and Egger’s regression test. The funnel plots demonstrated a generally symmetrical distribution, and Egger’s test results (allelic: *P* = 0.356; heterozygous: *P* = 0.670; dominant: *P* = 0.556) did not indicate significant evidence of publication bias (Figs. S5, S6).

## Discussion

The TP53 Arg72Pro, XRCC1 Arg399Gln, GSTP1 Ile105Val, and the GSTM3 indel exhibit minimal relation to the risk of cSCC under any genetic model.

### Association of TP53 polymorphism with susceptibility to cSCC

TP53 is the most commonly altered driver gene in cSCC [7]. The protein it encodes, p53, is an important tumor inhibitor by controlling cell cycle arrest, repairing DNA damage, maintaining genomic stability, and mediating cellular senescence and apoptosis [7, 37]. TP53 mutations in cSCC exhibit high heterogeneity, encompassing point mutations, missense mutations, and nonsense mutations. The most common polymorphism occurs at codon 72, where a G→C substitution results in the transformation from arginine (Arg) to proline (Pro) [38]. The two TP53 polymorphic variants display distinct functional properties [39]. Dumont et al. [40] reported that, compared to the Pro72 variant, the Arg72 variant demonstrates an enhanced ability to promote apoptosis.

The findings of this study suggest no marked link of the TP53 Arg72Pro polymorphism to cSCC across all models.

Subgroup analyses stratified by sample size and experimental methodology similarly revealed no statistically significant associations. Region-based subgroup analysis indicated that the TP53 polymorphism was not associated with an increased cSCC risk among Asian populations under the recessive genetic model. However, significant associations were observed under the remaining five genetic models. Subgroup analysis based on HWE status showed that in studies conforming to HWE, the polymorphism was not associated with increased cSCC risk under any genetic model. In contrast, studies deviating from HWE revealed a significant association under the additive and homozygous models, while no significant associations were observed under the other four models. It is important to note that only one study was conducted in an Asian population, comprising 106 cases and 200 controls. Similarly, only two studies deviated from HWE, with a total of 109 cases and 259 controls. The limited sample sizes in these subgroups may increase the likelihood of false-positive genetic associations due to insufficient statistical power. Our results align with findings from previous meta-analyses [41–44].

### Association of XRCC1 polymorphism with susceptibility to cSCC

XRCC1 encodes a scaffold protein influencing plenty of DNA damage restoration processes. It functions as a nucleating factor in the BER pathway, facilitating the assembly of DNA repair components at the damage site [45, 46]. A G→A substitution in exon 10 of XRCC1 causes the change to glutamine (Gln) from arginine (Arg) at codon 399, leading to the Arg399Gln polymorphism [47]. This polymorphism may alter BER efficiency, thereby influencing the genetic susceptibility to skin cancer [45, 46].

The findings of this study suggest no notable relation of the XRCC1 Arg399Gln polymorphism to the likelihood of cSCC in any genetic model. Subgroup analyses based on sample size and experimental methodology likewise revealed no statistically significant associations. Our findings are in concordance with prior meta-analyses [48, 49].

Subgroup analysis by geographic region demonstrated that the XRCC1 Arg399Gln polymorphism was associated with an increased risk of cSCC among European populations under the additive, allelic, and homozygous models. In contrast, a decreased risk was observed among North American populations under the additive, allelic, homozygous, and recessive models. However, it is important to note that only one study was conducted in Europe and two in North America. The potential for random error due to limited sample sizes cannot be disregarded, and larger studies are required to verify these preliminary findings.

Subgroup analysis based on the source of controls indicated that, in population-based studies, the XRCC1 Arg399Gln polymorphism was associated with a reduced risk of cSCC under the additive, allelic, homozygous, and recessive models. No significant associations were observed under the remaining two models. Hospital-based studies revealed no significant associations across all genetic models. The observed discrepancies are likely attributable to selection bias stemming from the use of different control sources. Population-based controls better reflect the genetic frequency of the source population, whereas hospital-based controls may introduce bias due to health-related factors, potentially obscuring true associations. Moreover, population-based studies generally involved larger sample sizes and exhibited lower heterogeneity, suggesting greater inter-study consistency and enhancing the credibility of the findings. In contrast, hospital-based studies showed higher heterogeneity, which may have masked true associations.

Our results underscore the necessity of conducting subgroup analyses based on control source and of routinely reporting control selection procedures in genetic association studies. Future well-designed case-control studies, with rigorously defined population-based controls, are warranted to confirm the current subgroup findings.

### Association of GSTP1 Ile105Val polymorphism and the GSTM3 indel with susceptibility to cSCC

GSTs represent a group of widely distributed isoenzymes whose main role involves facilitating the binding reaction between glutathione (GSH) and electrophilic compounds, thereby facilitating the detoxification and elimination of various potentially toxic chemicals, carcinogens, and lipophilic compounds [12]. GSTP1 catalyzes the conjugation of reduced glutathione with hydrophobic and electrophilic compounds, facilitating biotransformation and detoxification processes [50]. Similarly, GSTM3 is involved in cellular detoxification and the clearance of reactive oxygen species [51].

Several studies have explored the relation of GSTP1 Ile105Val and the GSTM3 indel to the likelihood of cSCC, yielding inconsistent results [31–34]. These discrepancies may be attributed to the relatively minor influence of these polymorphisms on cSCC risk or to the limited statistical power of individual studies. Therefore, a meta-analysis incorporating data from all pertinent studies was executed, thereby enhancing statistical power to further elucidate the roles of GSTP1 Ile105Val and the GSTM3 indel in cSCC development.

No evident association exists between the GSTP1 Ile105Val polymorphism and cSCC susceptibility in any genetic model.

Subgroup analysis based on the source of controls revealed no statistically significant association between the GSTP1 Ile105Val polymorphism and the risk of cSCC under any genetic model.

Subgroup analysis by geographic region indicated that the GSTP1 Ile105Val variant was associated with a decreased risk of cSCC among Europeans under the additive, allelic, and recessive models. However, under the homozygous model, the polymorphism was associated with an increased risk of cSCC in the same population. Notably, the 95% confidence intervals (CIs) across all genetic models were markedly wide, suggesting imprecision of effect estimates. Furthermore, only two studies were conducted in European populations, and the limited sample size may contribute to random error and exaggerated ORs. Additionally, the included studies did not adequately adjust for critical confounding factors such as UV exposure dose and skin phenotype, which could either obscure or inflate true associations. Further research is warranted in large-scale, multicenter European cohorts with standardized phenotypic and environmental variable assessments to validate these findings.

Although the overall analysis (comprising four studies, total sample size = 829 cases) did not identify a significant association between the GSTP1 Ile105Val variant and cSCC risk, subgroup analysis based on sample size revealed a significant inverse association in the allelic model among three studies with sample sizes < 200 (combined total = 491 cases). Given the low statistical power and high sampling variability inherent in small studies, these findings may represent false-positive results. The only study with a relatively large sample size (*n* = 338) contributed greater weight to the meta-analysis and demonstrated an opposite direction of effect, thereby attenuating the pooled association. Further large-scale, multicenter studies with rigorous control for multiple comparisons are essential for reliably assessing the role of the GSTP1 Ile105Val polymorphism in cSCC susceptibility.

No evident association was identified between the GSTM3 indel and susceptibility to SCC under any genetic model. Although all studies investigating this polymorphism were homogeneous in terms of control source and genotyping methodology, and showed no inter-study heterogeneity, they were limited to only three studies with a combined sample size of 673 participants. Subgrouping further reduced sample sizes per comparison group, likely resulting in insufficient power to detect modest but potentially meaningful genetic effects. It is recommended that future studies aim to increase statistical power by expanding sample sizes or conducting multicenter collaborative research.

Our findings on GSTP1 Ile105Val polymorphism are consistent with previous meta-analyses [48]. In addition, this meta-analysis, which is the first to specifically examine the association between the GSTM3 indel and susceptibility to cSCC, did not reveal any statistically significant correlation.

## Limitations

Our meta-analysis has limitations. (1) This study encompassed only 22 original studies. The limited quantity of incorporated studies could cause publication bias. Therefore, subsequent investigations should aim to include more qualifying studies, conduct thorough literature assessments, and implement rigorous statistical approaches. Notably, the meta-analysis of TP53 Arg72Pro polymorphism showed no statistically significant publication bias. (2) Regarding the subgroup analysis by geographic region, merely one study on TP53 Arg72Pro polymorphism was performed in Asians, whereas the rest focused on European and North American populations. Similarly, for the XRCC1 Arg399Gln polymorphism, two studies were conducted in an Asian population, with the rest focusing on European and North American populations. Moreover, no studies on GSTP1 Ile105Val and the GSTM3 indel were conducted in Asian populations. The lack of data from other geographic regions, such as Asia, Africa, and South America, limits the application of our findings. Additional studies with expanded sample sizes and wider geographical coverage would help verify our results. (3) Some of the included studies exhibited methodological shortcomings, which may have led to spurious findings in genetic association studies. For example, in the included studies, four reported deviations from Hardy-Weinberg equilibrium in the control group, which may be attributable to genotyping errors or selection bias within the control populations [24, 26, 30, 33]. Furthermore, inconsistencies in study design were observed across the included studies, with some being hospital-based and others population-based. Therefore, high-quality, well-designed studies are necessary to corroborate our conclusions. (4) Small sample sizes in some studies compromised their statistical power to establish valid links of TP53 Arg72Pro polymorphism to the disease risk. These limitations raise the possibility that the observed significant associations may be false-positive. (5) The unavailability of individual-level data, including genotype information and environmental risk factors, limited our ability to conduct further assessments of possible gene-gene and gene-environment interactions.

Despite the foregoing limitations, overall, TP53 Arg72Pro, XRCC1 Arg399Gln, GSTP1 Ile105Val, and the GSTM3 indel are unlikely to correlate significantly with cSCC risk. Patients with cSCC derive only modest benefits from surgery, chemotherapy, radiotherapy, and supportive care, and those with advanced disease often experience poor clinical outcomes. In the era of predictive biomarkers, a thorough comprehension of the molecular mechanisms behind cSCC and the identification of specific biomarkers that can predict cSCC risk and guide therapeutic decision-making may facilitate the discovery of novel drug targets and the development of more effective targeted treatments, ultimately increasing treatment options for patients with poor prognoses [4]. Therefore, further large-scale, well-designed research is warranted to unravel the impact of TP53 Arg72Pro, XRCC1 Arg399Gln, GSTP1 Ile105Val, and the GSTM3 indel on cSCC risk.

## Supplementary Information

Below is the link to the electronic supplementary material.

Supplementary Material 1.

Supplementary Material 2.

## Data Availability

All data supporting the findings of this study are available within the paper and its supplementary information files. The complete dataset generated and analysed during this systematic review and meta-analysis is provided as a Supplementary Data file (Supplementary Data.xlsx).
