# Supplementary material for: A study on the association between TP53 Arg72Pro, XRCC1 Arg399Gln, GSTP1 Ile105Val, and GSTM3 indel and the risk of cutaneous squamous cell carcinoma: a systematic review and meta-analysis
Source: Discov Oncol. 2026 Jan 21;17:305. doi: 10.1007/s12672-026-04433-2 (PMC12905026; doi:10.1007/s12672-026-04433-2)
Supplement: Supplementary file 1 — Supplementary Material 1. [file 12672_2026_4433_MOESM1_ESM.docx]

**Supplementary Materials**

**Supplementary Table**

**Supplementary Table 1** Search result record

| Pubmed | | |
| --- | --- | --- |
| # | Query | Results |
| 1 | "Cutaneous Squamous Cell Carcinoma"[Title/Abstract] OR "Squamous cell carcinoma of the skin"[Title/Abstract] OR "cSCC"[Title/Abstract] OR "Cutaneous Squamous Cell Carcinomas"[Title/Abstract] OR "Squamous cell carcinomas of the skin"[Title/Abstract] | 5590 |
| 2 | Genes[MeSH Terms] | 837643 |
| 3 | "chromosome gene"[Title/Abstract] OR "Cistron"[Title/Abstract] OR "Cistrons"[Title/Abstract] OR "Gene"[Title/Abstract] OR "gene type"[Title/Abstract] OR "genes"[Title/Abstract] OR "Genetic Material"[Title/Abstract] OR "Genetic Materials"[Title/Abstract] OR "nested genes"[Title/Abstract] | 2726501 |
| 4 | Polymorphism, Single Nucleotide[MeSH Terms] | 144158 |
| 5 | "polymorphism"[Title/Abstract] OR "polymorphisms"[Title/Abstract] OR "single nucleotide polymorphism"[Title/Abstract] OR "Single Nucleotide Polymorphisms"[Title/Abstract] OR "single nucleotide variant"[Title/Abstract] OR "single nucleotide variation"[Title/Abstract] OR "SNPs"[Title/Abstract] OR "SNP"[Title/Abstract] | 355963 |
| 6 | #2 OR #3 | 2996089 |
| 7 | #4 OR #5 | 384684 |
| 8 | #6 OR #7 | 3117429 |
| 9 | #1 AND #8 | 874 |
|  |  |  |

| Embase | | |
| --- | --- | --- |
| # | Query | Results |
| 1 | 'cutaneous squamous cell carcinoma':ti,ab,kw OR 'squamous cell carcinoma of the skin':ti,ab,kw OR 'cSCC':ti,ab,kw OR 'cutaneous squamous cell carcinomas':ti,ab,kw OR 'squamous cell carcinomas of the skin':ti,ab,kw | 12442 |
| 2 | 'gene'/exp | 1303525 |
| 3 | 'chromosome gene':ti,ab,kw OR 'cistron':ti,ab,kw OR 'cistrons':ti,ab,kw OR 'gene':ti,ab,kw OR 'gene type':ti,ab,kw OR 'genes':ti,ab,kw OR 'genetic material':ti,ab,kw OR 'genetic materials':ti,ab,kw OR 'nested genes':ti,ab,kw | 3376930 |
| 4 | 'single nucleotide polymorphism'/exp | 260125 |
| 5 | 'polymorphism':ti,ab,kw OR 'polymorphisms':ti,ab,kw OR 'single nucleotide polymorphism':ti,ab,kw OR 'single nucleotide polymorphisms':ti,ab,kw OR 'single nucleotide variant':ti,ab,kw OR 'single nucleotide variation':ti,ab,kw OR 'snps':ti,ab,kw OR 'snp':ti,ab,kw | 460913 |
| 6 | #2 OR #3 | 3703096 |
| 7 | #4 OR #5 | 539169 |
| 8 | #6 OR #7 | 3889955 |
| 9 | #1 AND #8 | 2012 |

| Cochrane Library | | |
| --- | --- | --- |
| # | Query | Results |
| 1 | ('Cutaneous Squamous Cell Carcinoma' OR 'Squamous cell carcinoma of the skin' OR 'cSCC' OR 'Cutaneous Squamous Cell Carcinomas' OR 'Squamous cell carcinomas of the skin'):ti,ab,kw | 1282 |
| 2 | MeSH descriptor: [Genes] explode all trees | 2525 |
| 3 | ('chromosome gene' OR 'Cistron' OR 'Cistrons' OR 'Gene' OR 'gene type' OR 'genes' OR 'Genetic Material' OR 'Genetic Materials' OR 'nested genes'):ti,ab,kw | 53879 |
| 4 | MeSH descriptor: [Polymorphism, Single Nucleotide] explode all trees | 2534 |
| 5 | ('polymorphism' OR 'polymorphisms' OR 'single nucleotide polymorphism' OR 'Single Nucleotide Polymorphisms' OR 'single nucleotide variant' OR 'single nucleotide variation' OR 'SNPs' OR 'SNP'):ti,ab,kw | 11474 |
| 6 | #2 OR #3 | 54355 |
| 7 | #4 OR #5 | 11474 |
| 8 | #6 OR #7 | 58943 |
| 9 | #1 AND #8 | 86 |

| Web of Science | | |
| --- | --- | --- |
| # | Query | Results |
| 1 | TS=(Cutaneous Squamous Cell Carcinoma OR Squamous cell carcinoma of the skin OR cSCC OR Cutaneous Squamous Cell Carcinomas OR Squamous cell carcinomas of the skin) and Preprint Citation Index | 36789 |
| 2 | TS=(chromosome gene OR Cistron OR Cistrons OR Gene OR gene type OR genes OR Genetic Material OR Genetic Materials OR nested genes) and Preprint Citation Index | 5846684 |
| 3 | TS=(polymorphism OR polymorphisms OR single nucleotide polymorphism OR Single Nucleotide Polymorphisms OR single nucleotide variant OR single nucleotide variation OR SNPs OR SNP) and Preprint Citation Index | 702806 |
| 4 | #2 OR #3 and Preprint Citation Index | 6032878 |
| 5 | #1 AND #4 and Preprint Citation Index | 7065 |

**Supplementary Table 2** Quality assessment of the included studies

| Gene | Author | Year | Study selection | | | | Comparability between groups | Measurement of exposure factors | | | Total points |
| --- | --- | --- | --- | --- | --- | --- | --- | --- | --- | --- | --- |
|  |  |  | Is the Case Definition Adequate? (1 point) | Representativeness of the Cases (1 point) | Selection of Controls (1 point) | Definition of Controls (1 point) |  | Definition of Controls (1) | The same method was used to determine the exposure factors in case and control groups (1) | Non-Response Rate (1) |  |
| TP53 Arg72Pro | Marshall | 2000 | 1 | 1 | 1 | 1 | 1 | 1 | 1 | 1 | 8 |
|  | Dokianakis | 2000 | 1 | 1 | 1 | 1 | 0 | 1 | 1 | 0 | 6 |
|  | Bastiaens | 2001 | 1 | 1 | 1 | 1 | 1 | 1 | 1 | 0 | 7 |
|  | Cairey-Remonnay | 2002 | 1 | 1 | 1 | 1 | 0 | 1 | 1 | 1 | 7 |
|  | McGregor | 2002 | 1 | 1 | 1 | 1 | 1 | 1 | 1 | 1 | 8 |
|  | Humbey | 2003 | 1 | 1 | 1 | 1 | 0 | 1 | 1 | 0 | 6 |
|  | Gustafsson | 2004 | 1 | 1 | 1 | 1 | 1 | 1 | 1 | 1 | 8 |
|  | Han | 2006 | 1 | 1 | 1 | 1 | 2 | 1 | 1 | 1 | 9 |
|  | Bendesky | 2007 | 1 | 1 | 1 | 1 | 1 | 1 | 1 | 0 | 7 |
|  | Almquist | 2011 | 1 | 1 | 1 | 1 | 2 | 1 | 1 | 1 | 9 |
|  | Loeb | 2012 | 1 | 1 | 1 | 1 | 2 | 1 | 1 | 1 | 9 |
|  | Pandith | 2012 | 1 | 1 | 1 | 1 | 2 | 1 | 1 | 0 | 8 |
| XRCC1 Arg399 Gln | Nelson | 2002 | 1 | 1 | 1 | 1 | 2 | 1 | 1 | 1 | 9 |
|  | Han | 2004 | 1 | 1 | 1 | 1 | 2 | 1 | 1 | 1 | 9 |
|  | Kang | 2007 | 1 | 1 | 1 | 1 | 2 | 1 | 1 | 0 | 8 |
|  | Chiyomaru | 2012 | 1 | 0 | 1 | 1 | 1 | 1 | 1 | 1 | 7 |
|  | Surdu | 2014 | 1 | 1 | 1 | 1 | 2 | 1 | 1 | 1 | 9 |
| GSTP1 Lle105Val  AND GSTM3 | Ramsay | 2001 | 1 | 1 | 0 | 1 | 2 | 1 | 1 | 1 | 8 |
|  | Fryer | 2004 | 1 | 1 | 1 | 1 | 2 | 1 | 1 | 1 | 9 |
|  | Lira | 2006 | 1 | 1 | 1 | 1 | 2 | 1 | 1 | 1 | 9 |
|  | Leite | 2007 | 1 | 1 | 1 | 1 | 2 | 1 | 1 | 0 | 8 |

**Supplementary Table 3** Subgroup Analysis of the Associations Between TP53 Arg72Pro, XRCC1 Arg399Gln, GSTP1 Ile105Val, and GSTM3 Polymorphisms and cSCC Risk Across Different Genetic Models

| SNPs | Genetic Model | SF | Group | number | Test of association | | Test of heterogeneity | | |
| --- | --- | --- | --- | --- | --- | --- | --- | --- | --- |
|  |  |  |  |  | OR (95 CI) | P-value | I^2^ (%) | P_h_ | Model |
| TP53 Arg72Pro | Additive |  |  | 9 | 1.046( 0.863-1.267) | 0.647 | 53.8 | 0.027 | R |
|  |  | Region | Asia | 1 | 1.599(1.141-2.240) | 0.006 | N | N | N |
|  |  |  | Europe | 4 | 0.959(0.660-1.394) | 0.826 | 44.0 | 0.148 | F |
|  |  |  | North America | 4 | 0.996( 0.811-1.224) | 0.973 | 43.7 | 0.149 | F |
|  |  | Sample size | <200 | 4 | 0.800(0.487-1.314) | 0.378 | 61.0 | 0.053 | R |
|  |  |  | ≥200 | 5 | 1.133( 0.953-1.346) | 0.156 | 41.4 | 0.145 | F |
|  |  | control source | PB | 6 | 0.941( 0.753-1.174) | 0.588 | 49.0 | 0.081 | R |
|  |  |  | HB | 3 | 1.319( 1.009-1.725) | 0.043 | 20.8 | 0.283 | F |
|  |  | assay | PCR-SSP | 2 | 0.961( 0.671-1.377) | 0.828 | 0.0 | 0.549 | F |
|  |  |  | GEA | 4 | 1.087( 0.770-1.535) | 0.635 | 71.1 | 0.016 | R |
|  |  |  | ST | 2 | 0.911(0.325-2.554) | 0.859 | 83.4 | 0.014 | R |
|  |  |  | TaqMan | 1 | 1.094( 0.874-1.370) | 0.433 | N | N | N |
|  |  | HWE | HWE-compliant group | 8 | 0.988( 0.832-1.174) | 0.891 | 34.7 | 0.151 | F |
|  | Allelic | Region | Asia | 1 | 1.622( 1.156-2.276) | 0.005 | N | N | N |
|  |  |  | Europe | 7 | 0.850( 0.604-1.196) | 0.350 | 48.6 | 0.069 | R |
|  |  |  | North America | 4 | 0.991( 0.800-1.229) | 0.938 | 47.7 | 0.125 | F |
|  |  | Sample size | <200 | 6 | 0.788( 0.494-1.258) | 0.318 | 53.5 | 0.057 | R |
|  |  |  | ≥200 | 6 | 1.059( 0.855-1.312) | 0.597 | 64.1 | 0.016 | R |
|  |  | control source | PB | 7 | 0.908( 0.711-1.160) | 0.441 | 52.7 | 0.048 | R |
|  |  |  | HB | 5 | 1.087( 0.740-1.597) | 0.671 | 68.9 | 0.012 | R |
|  |  | assay | PCR-SSP | 3 | 0.934(0.667-1.308) | 0.691 | 0.0 | 0.402 | F |
|  |  |  | GEA | 5 | 1.095( 0.783-1.531) | 0.598 | 65.7 | 0.020 | R |
|  |  |  | ST | 3 | 0.776( 0.402-1.500) | 0.451 | 77.4 | 0.012 | R |
|  |  |  | TaqMan | 1 | 1.093( 0.874-1.367) | 0.437 | N | N | N |
|  |  | HWE | HWE-compliant group | 10 | 0.931( 0.771-1.125) | 0.460 | 47.9 | 0.045 | R |
|  |  |  | HWE-non-compliant group | 2 | 0.866( 0.151-4.957) | 0.872 | 64.7 | 0.092 | R |
|  | Homozygous | Region | Asia | 1 | 2.137(1.040-4.392) | 0.039 | N | N | N |
|  |  |  | Europe | 4 | 0.935(0.348-2.511) | 0.893 | 49.7 | 0.114 | F |
|  |  |  | North America | 4 | 0.991(0.708-1.388) | 0.959 | 0.0 | 0.560 | F |
|  |  | Sample size | <200 | 4 | 0.705(0.229-2.174) | 0.543 | 55.1 | 0.083 | R |
|  |  |  | ≥200 | 5 | 1.188(0.881-1.604) | 0.259 | 0.0 | 0.488 | F |
|  |  | control source | PB | 6 | 0.911(0.557-1.490) | 0.711 | 36.8 | 0.161 | F |
|  |  |  | HB | 3 | 1.626(0.942-2.807) | 0.081 | 0.0 | 0.521 | F |
|  |  | assay | PCR-SSP | 2 | 0.972(0.391-2.420) | 0.952 | 0.0 | 0.683 | F |
|  |  |  | GEA | 4 | 1.044(0.530-2.054) | 0.901 | 58.9 | 0.063 | R |
|  |  |  | ST | 2 | 1.146(0.135-9.710) | 0.901 | 76.4 | 0.040 | R |
|  |  |  | TaqMan | 1 | 1.186(0.659-2.134) | 0.569 | N | N | N |
|  |  | HWE | HWE-compliant group | 8 | 0.972(0.683-1.383) | 0.875 | 12.9 | 0.329 | F |
|  |  |  | HWE-non-compliant group | 1 | 2.137(1.040-4.392) | 0.039 | N | N | N |
|  | Heterozygous | Region | Asia | 1 | 2.862(1.644-4.984) | 0.000 | N | N |  |
|  |  |  | Europe | 7 | 0.849(0.639-1.127) | 0.257 | 0.0 | 0.442 | F |
|  |  |  | North America | 4 | 1.004(0.752-1.343) | 0.976 | 50.0 | 0.112 | F |
|  |  | Sample size | <200 | 6 | 0.794(0.503-1.254) | 0.322 | 21.7 | 0.271 | F |
|  |  |  | ≥200 | 6 | 1.158(0.834-1.609) | 0.380 | 73.0 | 0.002 | R |
|  |  | control source | PB | 7 | 0.948(0.759-1.185) | 0.640 | 20.9 | 0.270 | F |
|  |  |  | HB | 5 | 1.287(0.713-2.322) | 0.402 | 76.5 | 0.002 | R |
|  |  | assay | PCR-SSP | 3 | 0.854(0.504-1.448) | 0.558 | 14.8 | 0.309 | F |
|  |  |  | GEA | 5 | 1.394(0.831-2.338) | 0.208 | 70.9 | 0.008 | R |
|  |  |  | ST | 3 | 0.696(0.387-1.251) | 0.226 | 53.8 | 0.115 | R |
|  |  |  | TaqMan | 1 | 1.099(0.823-1.467) | 0.522 | N | N | N |
|  |  | HWE | HWE-compliant group | 10 | 0.963(0.806-1.151) | 0.680 | 16.2 | 0.294 | F |
|  |  |  | HWE-non-compliant group | 2 | 0.837(0.047-14.789) | 0.903 | 80.8 | 0.022 | R |
|  | Dominant | Region | Asia | 1 | 2.651(1.564-4.494) | 0.000 | N | N | N |
|  |  |  | Europe | 7 | 0.831(0.580-1.192) | 0.315 | 33.7 | 0.171 | F |
|  |  |  | North America | 4 | 0.994(0.742-1.332) | 0.968 | 54.5 | 0.086 | R |
|  |  | Sample size | <200 | 6 | 0.748(0.443-1.266) | 0.280 | 44.9 | 0.106 | F |
|  |  |  | ≥200 | 6 | 1.136(0.824-1.566) | 0.438 | 73.9 | 0.002 | R |
|  |  | control source | PB | 7 | 0.900(0.682-1.188) | 0.457 | 44.7 | 0.093 | R |
|  |  |  | HB | 5 | 1.240(0.690-2.231) | 0.472 | 77.5 | 0.001 | R |
|  |  | assay | PCR-SSP | 3 | 0.826(0.458-1.489) | 0.525 | 28.1 | 0.249 | F |
|  |  |  | GEA | 5 | 1.281(0.771-2.129) | 0.340 | 73.1 | 0.005 | R |
|  |  |  | ST | 3 | 0.703(0.346-1.427) | 0.329 | 70.7 | 0.033 | R |
|  |  |  | TaqMan | 1 | 1.111(0.842-1.465) | 0.456 | N | N | N |
|  |  | HWE | HWE-compliant group | 10 | 0.935(0.756-1.157) | 0.536 | 38.2 | 0.104 | F |
|  |  |  | HWE-non-compliant group | 2 | 0.748(0.040-13.993) | 0.846 | 82.0 | 0.019 | R |
|  | Recessive | Region | Asia | 1 | 1.147(0.614-2.141) | 0.667 | N | N | N |
|  |  |  | Europe | 4 | 0.950(0.379-2.383) | 0.913 | 44.7 | 0.143 | F |
|  |  |  | North America | 4 | 0.988(0.711-1.374) | 0.945 | 0.0 | 0.829 | F |
|  |  | Sample size | <200 | 4 | 0.790(0.289-2.155) | 0.645 | 46.4 | 0.133 | F |
|  |  |  | ≥200 | 5 | 1.052(0.790-1.402) | 0.727 | 0.0 | 0.988 | F |
|  |  | control source | PB | 6 | 0.958(0.639-1.435) | 0.834 | 20.3 | 0.281 | F |
|  |  |  | HB | 3 | 1.100(0.671-1.803) | 0.705 | 0.0 | 0.962 | F |
|  |  | assay | PCR-SSP | 2 | 1.001(0.413-2.425) | 0.998 | 0.0 | 0.770 | F |
|  |  |  | GEA | 4 | 0.942(0.662-1.339) | 0.739 | 3.7 | 0.374 | F |
|  |  |  | ST | 2 | 1.311(0.242 -7.086) | 0.753 | 64.2 | 0.095 | R |
|  |  |  | TaqMan | 1 | 1.142(0.642-2.031) | 0.651 | N | N | N |
|  |  | HWE | HWE-compliant group | 8 | 0.982(0.731-1.319) | 0.904 | 0.0 | 0.503 | F |
|  |  |  | HWE-non-compliant group | 1 | 1.147(0.614-2.141) | 0.667 | N | N | N |
| XRCC1 Arg399Gln | Additive | Region | Asia | 2 | 1.063(0.717-1.576) | 0.761 | 30.9 | 0.229 | F |
|  |  |  | Europe | 1 | 1.475(1.024-2.124) | 0.037 | N | N | N |
|  |  |  | North America | 2 | 0.843(0.726-0.980) | 0.026 | 0.0 | 0.729 | F |
|  |  | Sample size | <200 | 1 | 0.818(0.466-1.435) | 0.484 | N | N | N |
|  |  |  | ≥200 | 4 | 1.024(0.794-1.322) | 0.854 | 70.1 | 0.018 | R |
|  |  | control source | PB | 2 | 0.843(0.726 -0.980) | 0.026 | 0.0 | 0.729 | F |
|  |  |  | HB | 3 | 1.214(0.902-1.633) | 0.201 | 32.8 | 0.226 | F |
|  |  | assay | PCR-RFLP | 3 | 0.933(0.705-1.233) | 0.624 | 43.4 | 0.171 | F |
|  |  |  | TaqMan | 2 | 1.103(0.654-1.862) | 0.713 | 84.3 | 0.011 | R |
|  | Allelic | Region | Asia | 2 | 1.044(0.657-1.657) | 0.856 | 38.4 | 0.202 | F |
|  |  |  | Europe | 1 | 1.461(1.021-2.091) | 0.038 | N | N | N |
|  |  |  | North America | 2 | 0.835(0.716-0.975) | 0.022 | 0.0 | 0.739 | F |
|  |  | Sample size | <200 | 1 | 0.751(0.385-1.465) | 0.401 | N | N | N |
|  |  |  | ≥200 | 4 | 1.020(0.787-1.321) | 0.882 | 70.6 | 0.017 | R |
|  |  | control source | PB | 2 | 0.835(0.716-0.975) | 0.022 | 0.0 | 0.739 | F |
|  |  |  | HB | 3 | 1.217(0.895-1.653) | 0.210 | 32.5 | 0.227 | F |
|  |  | assay | PCR-RFLP | 3 | 0.925 (0.682-1.255) | 0.616 | 47.4 | 0.150 | F |
|  |  |  | TaqMan | 2 | 1.094(0.648-1.847) | 0.735 | 84.5 | 0.011 | R |
|  | Homozygous | Region | Asia | 2 | 1.097(0.269-4.469) | 0.898 | 67.8 | 0.078 | R |
|  |  |  | Europe | 1 | 2.212(1.063-4.602) | 0.034 | N | N | N |
|  |  |  | North America | 2 | 0.668(0.479-0.931) | 0.017 | 0.0 | 0.435 | F |
|  |  | Sample size | <200 | 1 | 0.486(0.127-1.857) | 0.291 | N | N | N |
|  |  |  | ≥200 | 4 | 1.096(0.583-2.062) | 0.776 | 76.7 | 0.005 | R |
|  |  | control source | PB | 2 | 0.668(0.479-0.931) | 0.017 | 0.0 | 0.435 | F |
|  |  |  | HB | 3 | 1.537(0.706-3.347) | 0.279 | 50.4 | 0.133 | R |
|  |  | assay | PCR-RFLP | 3 | 0.846(0.342 -2.089) | 0.717 | 69.1 | 0.039 | R |
|  |  |  | TaqMan | 2 | 1.233(0.427-3.562) | 0.699 | 83.9 | 0.013 | R |
|  | Heterozygous | Region | Asia | 2 | 1.090(0.693-1.715) | 0.708 | 0.0 | 0.508 | F |
|  |  |  | Europe | 1 | 1.393(0.784-2.475) | 0.258 | N | N | N |
|  |  |  | North America | 2 | 0.914(0.733-1.140) | 0.427 | 0.0 | 0.536 | F |
|  |  | Sample size | <200 | 1 | 1.457(0.552-3.845) | 0.447 | N | N | N |
|  |  |  | ≥200 | 4 | 0.971(0.802-1.176) | 0.762 | 0.0 | 0.532 | F |
|  |  | control source | PB | 2 | 0.914(0.733-1.140) | 0.427 | 0.0 | 0.536 | F |
|  |  |  | HB | 3 | 1.198(0.839-1.709) | 0.320 | 0.0 | 0.648 | F |
|  |  | assay | PCR-RFLP | 3 | 1.024(0.783-1.341) | 0.860 | 0.0 | 0.759 | N |
|  |  |  | TaqMan | 2 | 1.025(0.650-1.615) | 0.916 | 53.4 | 0.143 | R |
|  | Dominant | Region | Asia | 2 | 1.095(0.718-1.669) | 0.673 | 0.0 | 0.754 | F |
|  |  |  | Europe | 1 | 1.561(0.908-2.684) | 0.107 | N | N | N |
|  |  |  | North America | 2 | 0.849(0.690-1.046) | 0.124 | 0.0 | 0.822 | F |
|  |  | Sample size | <200 | 1 | 0.971(0.410-2.300) | 0.947 | N | N | N |
|  |  |  | ≥200 | 4 | 0.985(0.772-1.256) | 0.903 | 39.0 | 0.178 | F |
|  |  | control source | PB | 2 | 0.849(0.690-1.046) | 0.124 | 0.0 | 0.822 | F |
|  |  |  | HB | 3 | 1.251(0.897-1.746) | 0.186 | 0.0 | 0.570 | F |
|  |  | assay | PCR-RFLP | 3 | 0.947(0.735-1.221) | 0.676 | 0.0 | 0.668 | F |
|  |  |  | TaqMan | 2 | 1.089(0.592-2.006) | 0.783 | 75.7 | 0.043 | R |
|  | Recessive | Region | Asia | 2 | 1.016(0.220-4.686) | 0.983 | 74.4 | 0.048 | R |
|  |  |  | Europe | 1 | 1.826(0.960-3.474) | 0.067 | N | N | N |
|  |  |  | North America | 2 | 0.697(0.503-0.966) | 0.030 | 6.9 | 0.300 | F |
|  |  | Sample size | <200 | 1 | 0.429(0.117-1.569) | 0.201 | N | N | N |
|  |  |  | ≥200 | 4 | 1.068(0.607-1.878) | 0.819 | 74.5 | 0.008 | R |
|  |  | control source | PB | 2 | 0.697(0.503-0.966) | 0.030 | 6.9 | 0.300 | F |
|  |  |  | HB | 3 | 1.377(0.639-2.970) | 0.414 | 54.9 | 0.109 | R |
|  |  | assay | PCR-RFLP | 3 | 0.821(0.328-2.055) | 0.674 | 72.2 | 0.027 | R |
|  |  |  | TaqMan | 2 | 1.166(0.524-2.595) | 0.707 | 77.4 | 0.035 | R |
| GSTP1 Ile105Val | Additive | Region | Europe | 1 | 0.558(0.332-0.937) | 0.027 | N | N | N |
|  |  |  | South America | 1 | 0.855(0.473-1.545) | 0.604 | N | N | N |
|  |  |  | Oceania | 1 | 1.028(0.747-1.414) | 0.865 | N | N | N |
|  |  | Sample size | <200 | 2 | 0.674(0.445-1.020) | 0.062 | 11.6 | 0.288 | F |
|  |  |  | ≥200 | 1 | 1.028(0.747-1.414) | 0.865 | N | N | N |
|  |  | control source | PB | 1 | 0.855(0.473-1.545) | 0.604 | N | N | N |
|  |  |  | HB | 2 | 0.785(0.433-1.422) | 0.424 | 74.2 | 0.049 | R |
|  | Allelic | Region | Europe | 2 | 0.535(0.345-0.831) | 0.005 | 0.0 | 0.876 | F |
|  |  |  | South America | 1 | 0.839(0.450-1.566) | 0.581 | N | N | N |
|  |  |  | Oceania | 1 | 1.030(0.741-1.431) | 0.860 | N | N | N |
|  |  | Sample size | <200 | 3 | 0.621(0.434-0.890) | 0.009 | 0.0 | 0.507 | F |
|  |  |  | ≥200 | 1 | 1.030(0.741-1.431) | 0.860 | N | N | N |
|  |  | control source | PB | 1 | 0.839(0.450-1.566) | 0.581 | N | N | N |
|  |  |  | HB | 3 | 0.713(0.433-1.174) | 0.184 | 63.6 | 0.064 | R |
|  | Homozygous | Region | Europe | 1 | 10.759(1.381-83.829) | 0.023 | N | N | N |
|  |  |  | South America | 1 | 0.476(0.099-2.291) | 0.355 | N | N | N |
|  |  |  | Oceania | 1 | 1.159(0.583-2.305) | 0.674 | N | N | N |
|  |  | Sample size | <200 | 2 | 2.104(0.099-44.530) | 0.633 | 82.1 | 0.018 | R |
|  |  |  | ≥200 | 1 | 1.159(0.583-2.305) | 0.674 | N | N | N |
|  |  | control source | PB | 1 | 0.476(0.099-2.291) | 0.355 | N | N | N |
|  |  |  | HB | 2 | 2.838(0.334-24.148) | 0.340 | 75.4 | 0.044 | R |
|  | Heterozygous | Region | Europe | 2 | 0.860(0.491-1.507) | 0.598 | 0.0 | 0.714 | F |
|  |  |  | South America | 1 | 1.211(0.519-2.825) | 0.658 | N | N | N |
|  |  |  | Oceania | 1 | 0.912(0.569-1.463) | 0.702 | N | N | N |
|  |  | Sample size | <200 | 3 | 0.955(0.598-1.524) | 0.846 | 0.0 | 0.752 | F |
|  |  |  | ≥200 | 1 | 0.912(0.569-1.463) | 0.702 | N | N | N |
|  |  | control source | PB | 1 | 1.211(0.519-2.825) | 0.658 | N | N | N |
|  |  |  | HB | 3 | 0.890(0.620-1.278) | 0.528 | 0.0 | 0.924 | F |
|  | Dominant | Region | Europe | 2 | 0.634(0.367-1.095) | 0.102 | 0.0 | 0.894 | F |
|  |  |  | South America | 1 | 1.004(0.447-2.256) | 0.992 | N | N | N |
|  |  |  | Oceania | 1 | 0.966(0.621-1.502) | 0.878 | N | N | N |
|  |  | Sample size | <200 | 3 | 0.732(0.466-1.151) | 0.177 | 0.0 | 0.648 | F |
|  |  |  | ≥200 | 1 | 0.966(0.621-1.502) | 0.878 | N | N | N |
|  |  | control source | PB | 1 | 1.004(0.447-2.256) | 0.992 | N | N | N |
|  |  |  | HB | 3 | 0.818(0.580-1.153) | 0.251 | 0.0 | 0.497 | F |
|  | Recessive | Region | Europe | 1 | 0.096(0.013-0.720) | 0.023 | N | N | N |
|  |  |  | South America | 1 | 0.436(0.095-1.999) | 0.285 | N | N | N |
|  |  |  | Oceania | 1 | 1.211(0.632-2.320) | 0.564 | N | N | N |
|  |  | Sample size | <200 | 2 | 0.238(0.056-1.017) | 0.053 | 27.5 | 0.240 | F |
|  |  |  | ≥200 | 1 | 1.211(0.632-2.320) | 0.564 | N | N | N |
|  |  | control source | PB | 1 | 0.436(0.095-1.999) | 0.285 | N | N | N |
|  |  |  | HB | 2 | 0.411(0.035-4.796) | 0.478 | 81.8 | 0.019 | R |
| GSTM3 | Additive | Sample size | <200 | 2 | 0.877(0.531-1.449) | 0.608 | 0.0 | 0.942 | F |
|  |  |  | ≥200 | 1 | 1.007(0.647-1.568) | 0.975 | N | N | N |
|  | Allelic | Sample size | <200 | 2 | 0.870(0.520-1.457) | 0.597 | 0.0 | 0.976 | F |
|  |  |  | ≥200 | 1 | 1.007(0.653-1.553) | 0.975 | N | N | N |
|  | Homozygous | Sample size | <200 | 2 | 1.599(0.461-5.549) | 0.460 | 0.0 | 0.528 | F |
|  |  |  | ≥200 | 1 | 0.819(0.147-4.561) | 0.820 | N | N | N |
|  | Heterozygous | Sample size | <200 | 2 | 0.647(0.334-1.252) | 0.196 | 0.0 | 0.811 | F |
|  |  |  | ≥200 | 1 | 1.042(0.634-1.713) | 0.871 | N | N | N |
|  | Dominant | Sample size | <200 | 2 | 0.739(0.403-1.357) | 0.330 | 0.0 | 0.873 | F |
|  |  |  | ≥200 | 1 | 1.027(0.631-1.671) | 0.915 | N | N | N |
|  | Recessive | Sample size | <200 | 2 | 1.785(0.520-6.128) | 0.357 | 0.0 | 0.506 | F |
|  |  |  | ≥200 | 1 | 0.810(0.146-4.489) | 0.809 | N | N | N |

Abbreviations: SNP, single nucleotide polymorphism; N, not available; OR, odds ratio; CI, confidence interval; F, fixed effect model; R, random-effect model; Ph, P-value of heterogeneity; SF, Stratification Factors; HWE, Hardy-Weinberg Equilibrium;PB, population-based; HB, hospital-based; GEA, Gel Electrophoresis Analysis, including PCR-DGGE and PCR-DGGE; ST, Sequencing Technology, Including AS-PCR-seq, nPCR-PSQ and nPCR-cDNA-seq.

**Supplementary Figures**


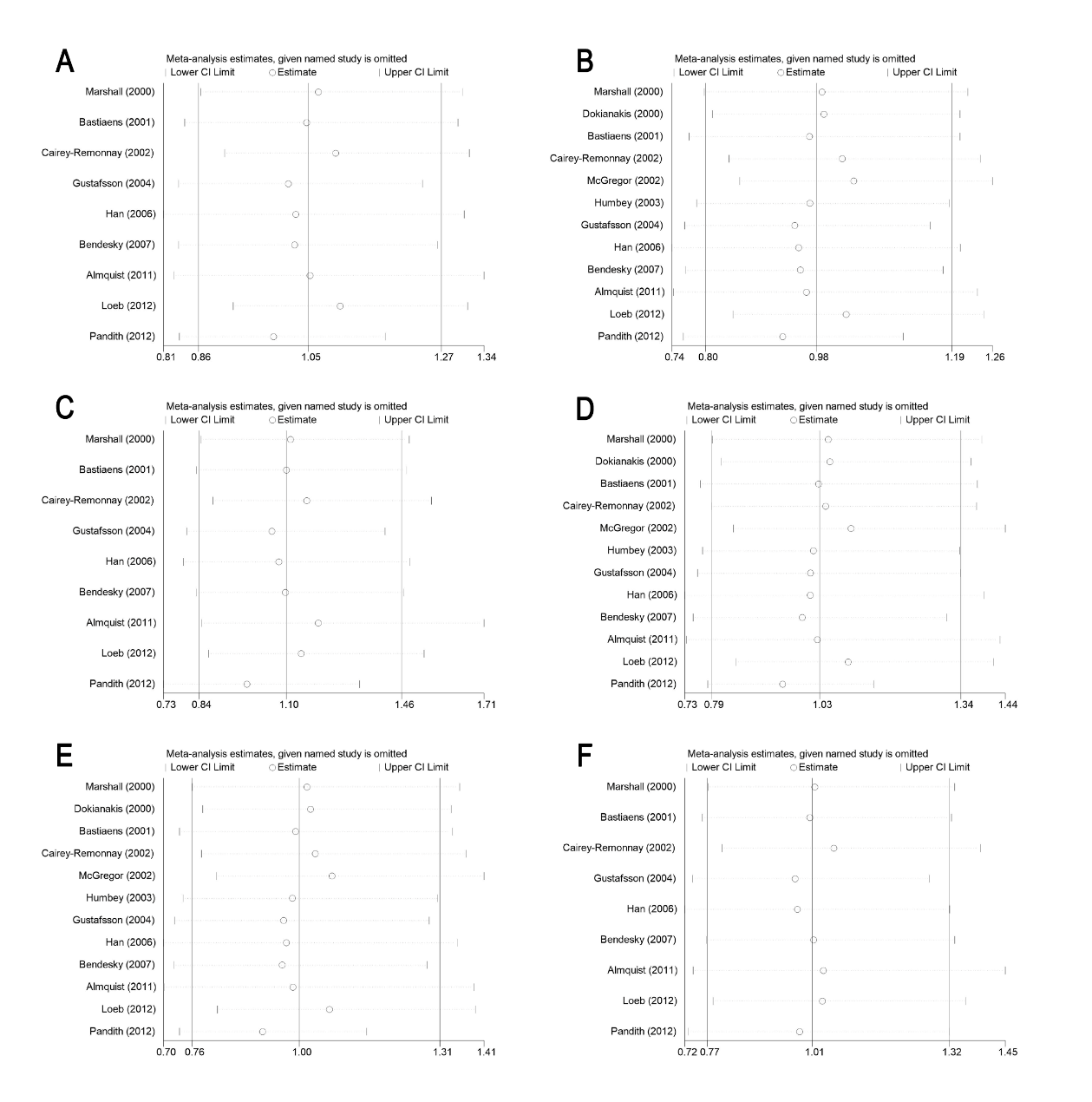


**Supplementary Figure 1** Forest plot of sensitivity analysis of the TP53 Arg72Pro

Note: A: Forest plot of sensitivity analysis of the TP53 Arg72Pro under the additive model; B: Forest plot of sensitivity analysis of the TP53 Arg72Pro under the allele model; C: Forest plot of sensitivity analysis of the TP53 Arg72Pro under the homozygote model; D: Forest plot of sensitivity analysis of the TP53 Arg72Pro under the heterozygote model; E: Forest plot of sensitivity analysis of the TP53 Arg72Pro under the dominant model; F: Forest plot of sensitivity analysis of the TP53 Arg72Pro under the recessive model


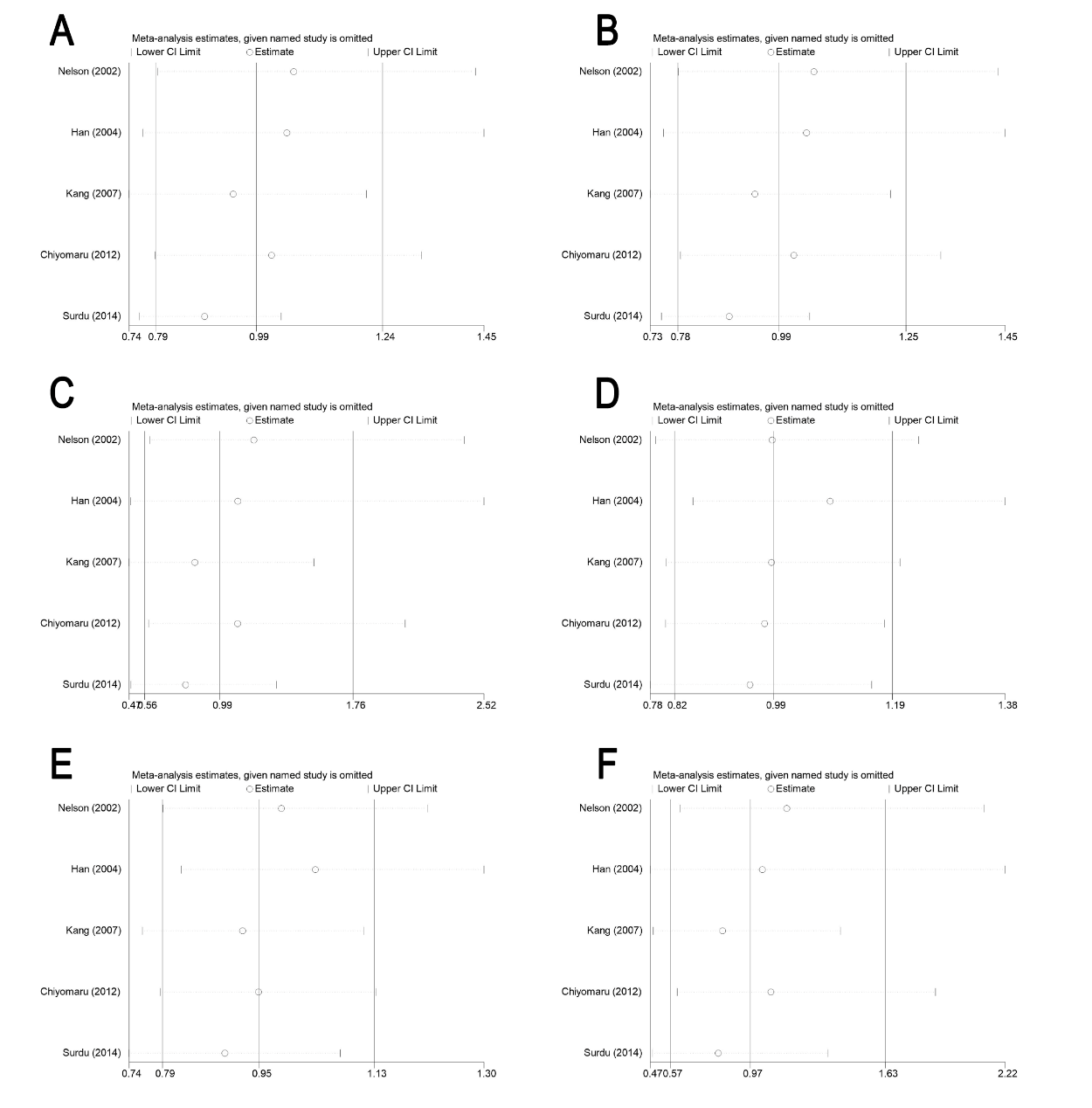


**Supplementary Figure 2** Forest plot of sensitivity analysis of the XRCC1 Arg399Gln

Note: A: Forest plot of sensitivity analysis of the XRCC1 Arg399Gln under the additive model; B: Forest plot of sensitivity analysis of XRCC1 Arg399Gln under the allele model; C: Forest plot of sensitivity analysis of the XRCC1 Arg399Gln under the homozygote model; D: Forest plot of sensitivity analysis of the XRCC1 Arg399Gln under the heterozygote model; E: Forest plot of sensitivity analysis of the XRCC1 Arg399Gln under the dominant model; F: Forest plot of sensitivity analysis of the XRCC1 Arg399Gln under the recessive model.


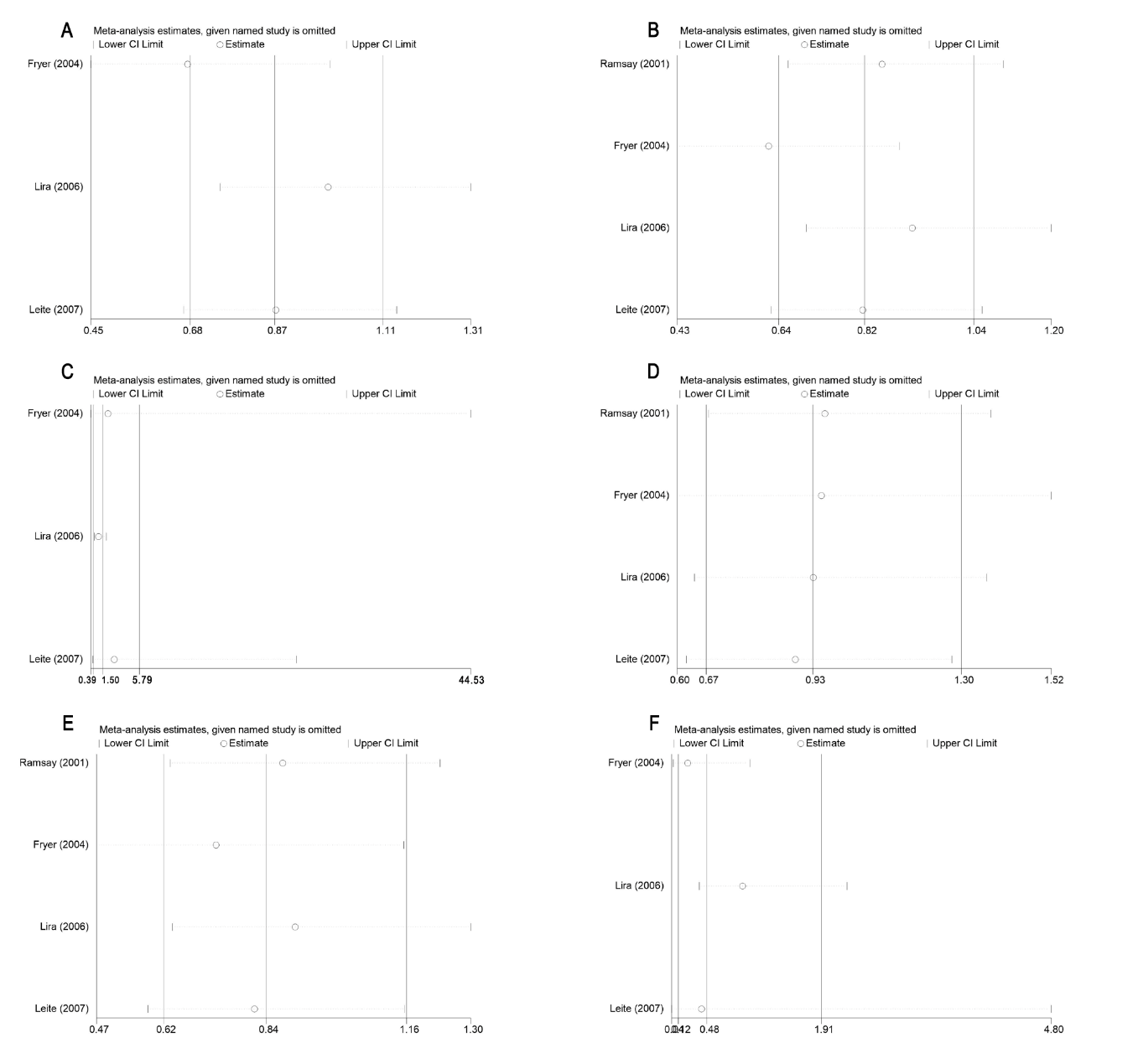


**Supplementary Figure 3** Forest plot of sensitivity analysis of GSTP1 Ile105Val.

Note: A: Forest plot of sensitivity analysis of GSTP1 Ile105Val under the additive model; B: Forest plot of sensitivity analysis of GSTP1 Ile105Val under the allele model; C: Forest plot of sensitivity analysis of GSTP1 Ile105Val under the homozygote model; D: Forest plot of sensitivity analysis of GSTP1 Ile105Val under the heterozygote model; E: Forest plot of sensitivity analysis of GSTP1 Ile105Val under the dominant model; F: Forest plot of sensitivity analysis of GSTP1 Ile105Val under the recessive model


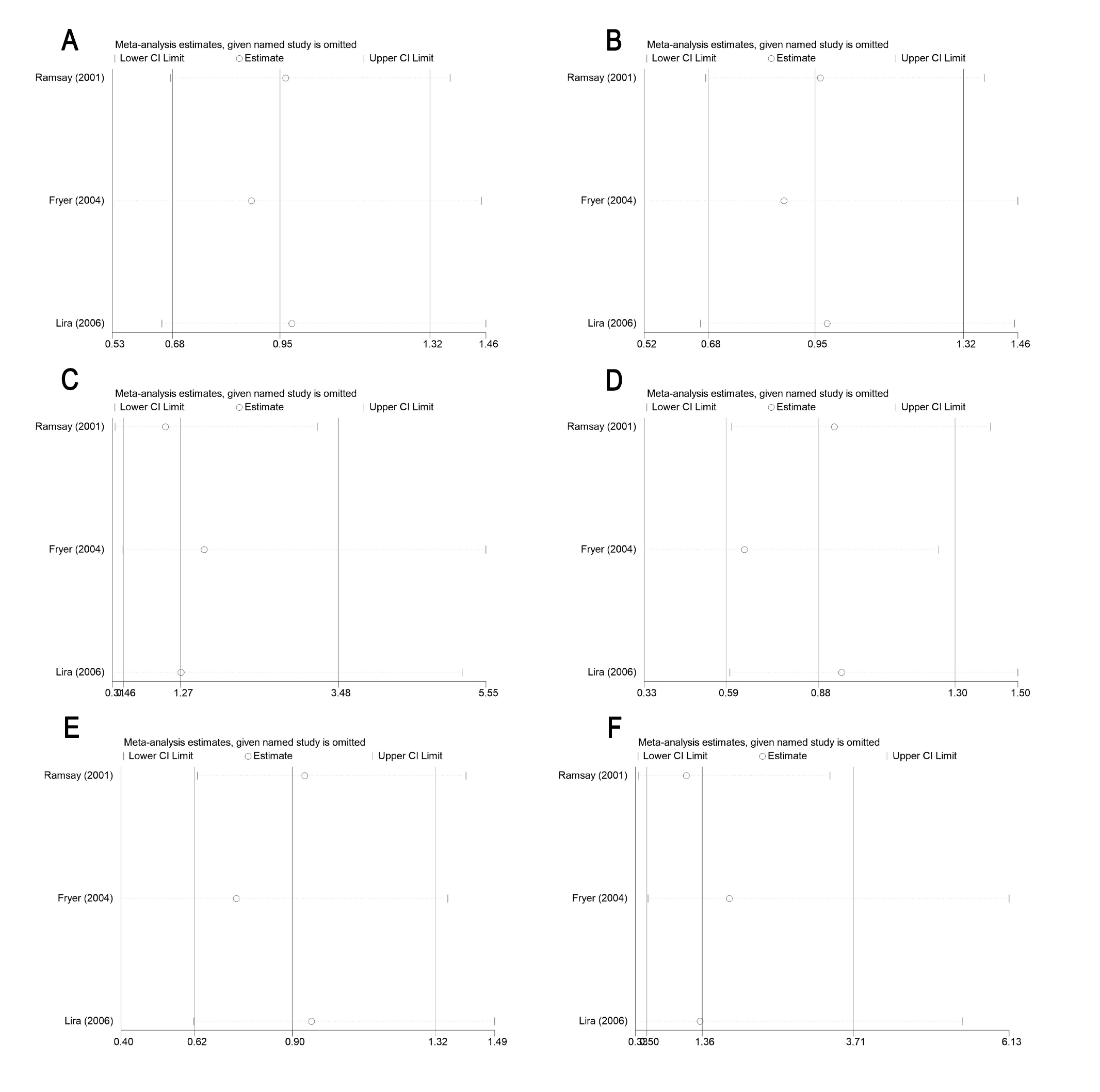


**Supplementary Figure 4** Forest plot of sensitivity analysis of the GSTM3

Note: A: Forest plot of sensitivity analysis of the GSTM3 under additive model; B: Forest plot of sensitivity analysis of the GSTM3 under allele model; C: Forest plot of sensitivity analysis of the GSTM3 under homozygote model; D: Forest plot of sensitivity analysis of the GSTM3 under heterozygote model; E: Forest plot of sensitivity analysis of GSTM3 under dominant model; F: Forest plot of sensitivity analysis of GSTM3 under recessive model.


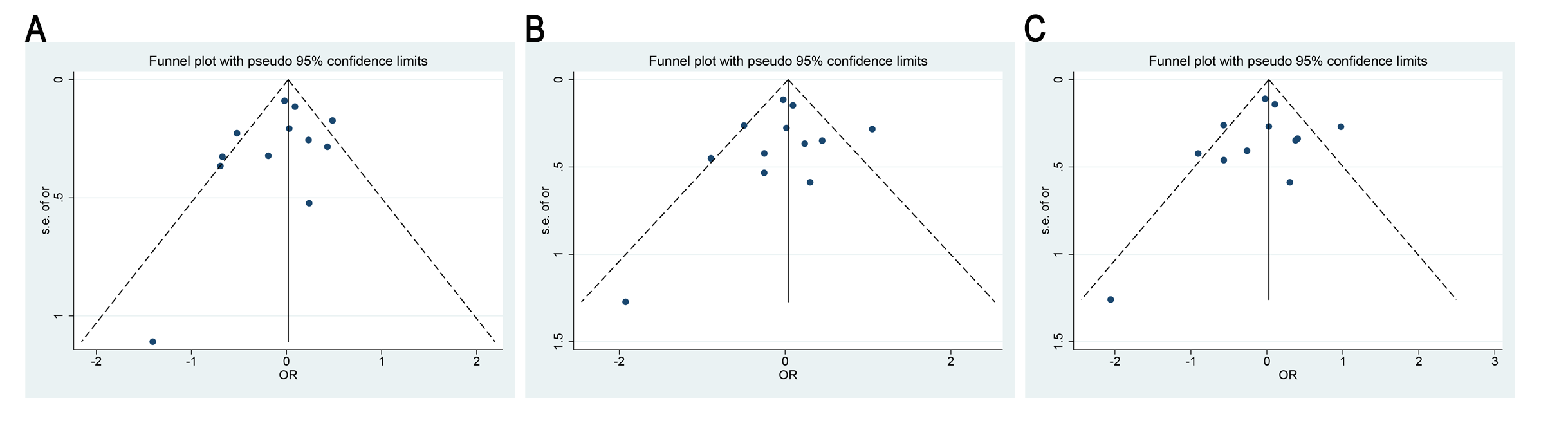


**Supplementary Figure 5** Funnel plot of publication bias

Note: A: Funnel plot of publication bias of TP53 Arg72Pro under allele model; B: Funnel plot of publication bias of TP53 Arg72Pro under heterozygote model; C: Funnel plot of publication bias of TP53 Arg72Pro under dominant model.

**
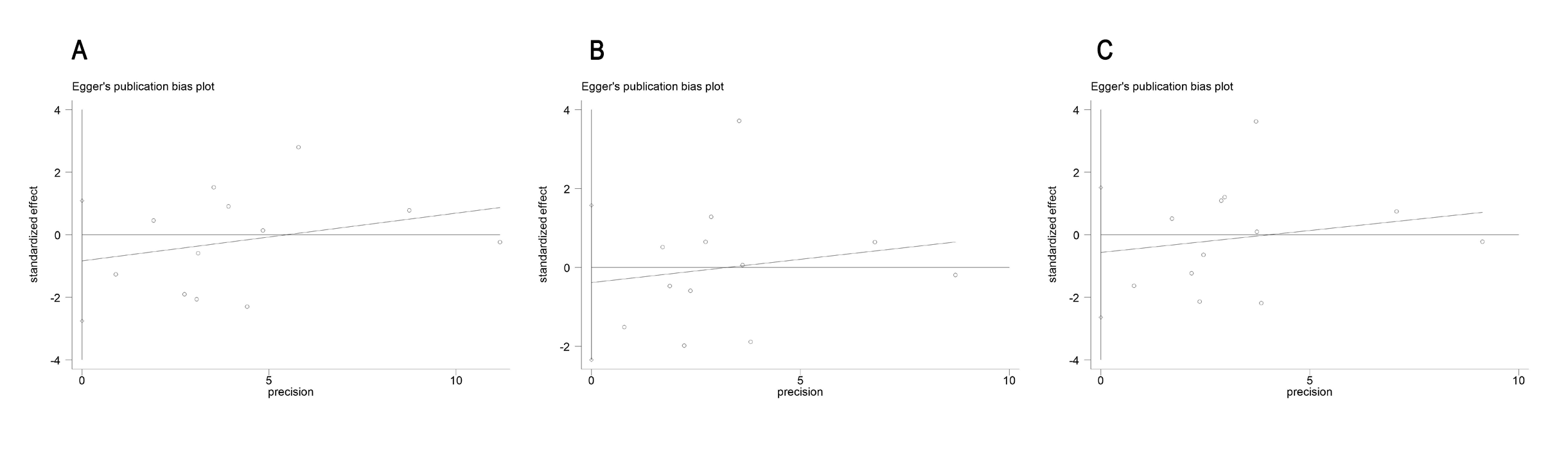
**

**Supplementary Figure 6** Egger’s test of TP53 Arg72Pro

Note: A: Egger’s test of TP53 Arg72Pro under the allele model; B: Egger’s test of TP53 Arg72Pro under in the heterozygote model; C: Egger’s test of TP53 Arg72Pro under in the dominant model
